# Efficient Predictor for Immunotherapy Efficacy: Detecting Pan‐Clones Effector Tumor Antigen‐Specific T Cells in Blood by Nanoparticles Loading Whole Tumor Antigens

**DOI:** 10.1002/advs.202409913

**Published:** 2024-11-05

**Authors:** Weibiao Zeng, Jin Wang, Zhike Chen, Jian Yang, Ao Zhu, Yan Zheng, Xianlan Chen, Yuhan Liu, Leilei Wu, Yufeng Xie, Sheng Ju, Jun Chen, Cheng Ding, Chang Li, Xin Tong, Mi Liu, Jun Zhao

**Affiliations:** ^1^ Institute of Thoracic Surgery The First Affiliated Hospital of Soochow University Soochow University Suzhou Jiangsu 215123 P. R. China; ^2^ Department of Pharmaceutics College of Pharmaceutical Sciences Soochow University Suzhou Jiangsu 215123 P. R. China; ^3^ Department of Thoracic Surgery, The First Affiliated Hospital of Soochow University Soochow University Suzhou Jiangsu 215123 P. R. China; ^4^ Department of Thoracic Surgery Shanghai General Hospital Shanghai Jiaotong University School of Medicine Shanghai 200080 P. R. China; ^5^ Institute of Minimally Invasive Thoracic Cancer Therapy and Translational Research Soochow University Suzhou Jiangsu 215123 P. R. China; ^6^ Department of Radiotherapy Shanghai Pulmonary Hospital of Tongji University Shanghai 200000 P. R. China; ^7^ Suzhou Ersheng Biopharmaceutical Co., Ltd Suzhou 215123 P. R. China; ^8^ Jiangsu Province Engineering Research Center of Precision Diagnostics and Therapeutics Development Soochow University Suzhou 215123 P. R. China; ^9^ Wuxi Boston Biopharmaceutical Co., Ltd Wuxi 214125 P. R. China

**Keywords:** antigen‐specific T cells, biomarker, cancer, immunotherapy, tumor antigen

## Abstract

Cancer involves tumor cells and tumor‐specific immunity. The ability to accurately quantify tumor‐specific immunity is limited. Most immunotherapies function by activating new effector tumor antigen‐specific T cells (ETASTs) or reactivating the pre‐existing ETASTs repertoire. Therefore, the amount of ETASTs can be used to characterize immunotherapy efficacy. Tumor antigens are highly heterogeneous and detecting most ETASTs is challenging. Therefore, nanoparticles loading whole‐cell tumor antigens are used to activate and detect pan‐clones ETASTs in the blood. The differences between ETASTs and other T cells are transformed into activated and non‐activated states. By measuring markers of the activated status and cytotoxic function of ETASTs, it can distinguish ETASTs from other T cells. ETASTs in patients with lung cancer are higher than those in healthy individuals and those with benign pulmonary nodules. Therapeutic efficacy positively correlated with the number of ETASTs in the blood. ETATS levels increase only in the blood of patients who respond to immunotherapy. Single‐cell sequencing studies validated these findings. This study provides a highly accurate, specific, non‐invasive, and efficient biomarker for predicting immunotherapy efficacy in lung and other cancers. This method can also be applied to evaluate the efficacy of other treatments, such as radiotherapy, oncolytic viruses, and nanomedicine‐based therapies.

## Graphical abstract

Effector tumor antigen antigen‐specific T cells (ETASTs) are the major cells of killing cancer cells. The amount of ETASTs reflects the level of tumor‐specific immune responses in tumor‐bearing body. Therefore, detecting ETASTs peripheral in blood of tumor‐bearing body by nanoparticles loading whole tumor antigens can be utilized to predict the therapeutic efficacy of cancer immunotherapy.

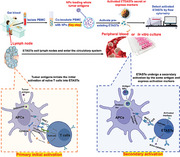

## 1 Introduction

Lung cancer, including adenocarcinoma and squamous cell carcinoma, is among the most prevalent and deadly malignancies worldwide, with non‐small cell lung cancer (NSCLC)^[^ ^1^ ^]^ being the predominant type. Traditional treatments such as surgery, chemotherapy, and radiotherapy often exhibit limited efficacy in improving the prognosis of NSCLC. The rapid development of immune checkpoint inhibitors (ICIs) in recent years has resulted in novel therapies for NSCLC. ICIs primarily block the interaction between inhibitory receptors on T cells and their corresponding ligands, modulating immune cell activity and reactivating and expanding pre‐existing tumor‐specific T cell subpopulations.^[^ ^2^, ^3^, ^4^ ^]^ Currently, the mainstay of NSCLC treatment involves inhibiting programmed cell death protein 1 (PD‐1) or programmed cell death ligand 1 (PD‐L1), alone or in combination with chemotherapy, which is now a primary treatment modality foradvanced patients with NSCLC.^[^ ^5^ ^]^ However, ICI therapy only benefits a subset of patients with NSCLC, and a considerable portion fails to derive clinical benefits. Consequently, identifying efficient predictive biomarkers for immunotherapy response is crucial.

Cancer is the result of a conflict between cancer cells and the immune system. The reported biomarkers to date have focused on evaluating the properties of cancer cells, including PD‐L1 expression levels,^[^ ^6^, ^7^, ^8^ ^]^ tumor mutation burden (TMB),^[^ ^9^, ^10^ ^]^ tumor‐infiltrating lymphocyte (TIL) counts,^[^ ^11^ ^]^ and neoantigen load.^[^ ^12^, ^13^ ^]^ No biomarkers have been systematically used to evaluate tumor‐specific immune responses in tumor‐bearing bodies. Assessing and presenting information on both tumor cells and tumor‐specific immunity would greatly benefit doctors and patients.

Despite the FDA approval to use PD‐L1 expression levels in NSCLC tumor tissues to select patients for ICI therapy, the discriminatory efficacy of this biomarker has not met expectations. The failure of the biomarker's efficacy implies that relying solely on PD‐L1 expression levels as a selection criterion may result in some patients not benefiting from ICI therapy or incorrectly excluding those who could have benefited.^[^ ^14^, ^15^ ^]^ Additionally, specific subsets of T cells within tumor tissues, known as tumor antigen‐specific T cells (TAST), have been shown in multiple studies to be predictive biomarkers of immunotherapy response.^[^ ^16^, ^17^ ^]^ These T cells can recognize tumor antigens specifically, but not all have cytotoxic functions after antigen recognition. Specific recognition of tumor antigens can be considered structurally specific, whereas the ability to kill cancer cells containing the antigen after recognition can be considered functionally specific. Based on structural and functional specificity, TAST can be classified into three categories: 1) Effector tumor antigen‐specific T cells (ETAST), capable of both specifically recognizing and killing cancer cells containing the antigen; 2) Regulatory tumor antigen‐specific T cells (RTASTs), which not only recognize tumor antigens specifically but also inhibit the function of ETASTs after recognition, thereby promoting tumor cell proliferation; and 3) Anergy tumor antigen‐specific T cells (ATASTs), which recognize tumor antigens specifically but lack cytotoxicity against cancer cells after recognition. Two recent studies demonstrated a positive correlation between the types and quantities of ETASTs in the body or peripheral blood of patients with cancer post‐immunotherapy and their prognosis.^[^ ^18^, ^19^ ^]^ ETASTs are the key force in killing cancer cells and are considered the true participants in ICIs therapy.^[^ ^20^, ^21^ ^]^ A successful ICIs therapy response relies on the reactivation of ETASTs.^[^ ^22^, ^23^ ^]^ Detection of ETASTs in peripheral blood could be an ideal biomarker to reflect immunotherapy efficacy.

Accurate detection of pan‐clone ETASTs in the peripheral blood is one of the best ways to assess tumor‐specific immunity in patients, but this remains a significant challenge. There are thousands of different tumor antigens, and one clone of TAST can recognize only one tumor antigen; thus, there are thousands of different clones of TASTs. According to the tumor‐immune cycle theory,^[^ ^24^ ^]^ after tumor antigens activate TASTs in draining lymph nodes, TASTs enter tumor tissues through the bloodstream, making blood the optimal source for detecting ETASTs. However, ETASTs in the blood are rare and highly heterogeneous.^[^ ^25^, ^26^ ^]^ Due to the highly heterogeneous nature of tumor antigens, it is challenging to detect all ETASTs accurately using ELISPOT or tetramer technology. ETASTs are not different from other T cells, except for the TCR recognizing different antigen peptides; therefore, distinguishing ETASTs from other T cells is very difficult, particularly when identifying all different clones of ETASTs. Currently, detecting a few well‐defined clones of ETASTs is mainly done through multimer technology or ELISPOT, which only detects T cells recognizing specific antigens. However, these methods have limited peptide antigens available for detection, low accuracy, and high costs, making their widespread implementation in clinical practice challenging. There is an urgent need for non‐invasive, facile, and efficacious peripheral blood ETASTs detection methods.

The highly heterogeneous nature of tumor cells and tumor antigens makes it difficult to detect pan‐clones (all or most clones) of ETASTs. Currently, no effective method exists to comprehensively and accurately detect broad ETAST clones in the blood, providing doctors with accurate information on tumor‐specific immune responses in patients with cancer. This study proposes that pan‐clone ETASTs in peripheral blood mononuclear cells (PBMCs) can be efficiently identified using nanoparticles (NPs) loaded with whole tumor antigens, thereby providing accurate information on tumor‐specific immune responses to doctors and patients with cancer. When co‐incubated with PBMCs, NPs are phagocytosed by the antigen‐presenting cells (APCs) in PBMC, releasing the encapsulated tumor antigens. The released antigens undergo degradation into antigen peptides on the surface APCs. Subsequently, antigen peptides presented on the surface of APCs can secondarily activate pre‐existing TASTs, which recognize the antigen originally activated by tumor antigens in draining lymph nodes. Dendritic cells (DCs) are the only APCs capable of activating naïve T cells in lymph nodes, while B cells, macrophages, and DCs serve as APCs for secondary activation of pre‐existing non‐naïve T cells in peripheral tissues^[^ ^27^ ^]^ or in vitro (Figure S1, Supporting Information). When Naïve T cells are primarily activated by tumor antigens through APCs and transformed into ETASTs in draining lymph nodes, all three signals —antigen peptide (signal 1), co‐stimulatory molecule (signal 2), and cytokine in the microenvironment (signal 3) — are required for the initial activation. However, only signal 1 is necessary when pre‐existing ETASTs, initially activated by tumor antigens, encounter and are reactivated by corresponding antigens in vitro or in the blood.

**Figure 1:**
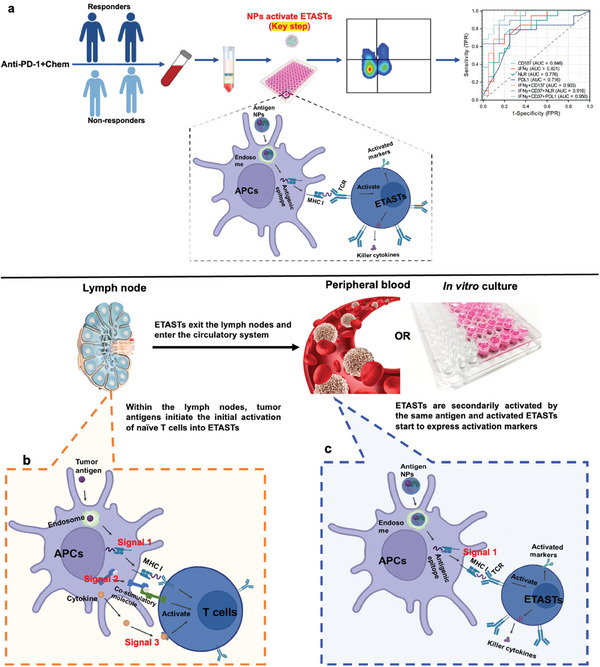
Schematic illustration of activation‐based pan‐clones ETAST detection in PBMC and mechanism of secondary activation of ETAST in peripheral or in vitro using nanoparticles loaded with whole tumor antigens. a) The Process of predicting immunotherapy efficacy by detecting ETASTs in PBMCs: PBMCs are isolated from the blood samples of cancer patients before and during treatment, followed by in vitro activation of ETASTs using NPs loaded with whole‐cell antigens (TATAN). All existing ETASTs clones are activated by their corresponding tumor antigens, and the activated ETASTs are detected by measuring activation biomarkers via flow cytometry (an increase in ETASTs after treatment indicates better therapeutic efficacy). b) Naïve T cells are primarily activated by tumor antigens through APCs (tumor antigens are phagocytosed by APCs and presented on the surface of APCs), transforming into TAST in draining lymph nodes. All three signals are required for the initial activations of TAST. Upon encountering tumor antigens in vitro or in the blood (Nanoparticles loaded with tumor antigens are phagocytosed by APCs, processed, and presented on the surface of APCs), pre‐existing TAST, which has been initially activated, will undergo secondary activation. Only signal 1 is required for secondary activation. NP, nanoparticle; NSCLC, non‐small cell lung cancer; ETAST, effector tumor antigen‐specific T cells; APCs, and antigen‐presenting cells.

This study defines nanoparticles loaded with whole tumor antigens as **T**umor **A**ntigen‐specific **T** cells **A**ctivating **N**anoparticles (TATAN). In this study, the activation of T cells in vitro using nanoparticles loaded with whole‐cell tumor antigens was an indirect process via autologous APCs, mimicking the activation of pre‐existing TASTs in the periphery after APC phagocytosis of in vivo antigens. T cells (ETASTs) that recognize tumor antigens are activated in vitro upon encountering these antigens, followed by the detection of activated ETASTs. Thus, the structural difference between ETASTs and other T cells is reflected in their activated status, allowing ETASTs to be distinguished by detecting activation biomarkers, such as IFN‐γ and CD137. Whether NPs loaded with whole tumor antigens are better than those loaded with multiple peptides or free tumor lysates for such activation needs to be explored. Importantly, universally activating NPs needs exploration for practical use. Thus, it is necessary to investigate whether NPs loaded with mixed lysates from multiple allogeneic tumor tissues or multiple homogeneous cancer cell lines can achieve the same activation and detection efficacy as NPs loaded with autologous tumor tissue lysates. The feasibility of using these NPs for activation‐based detection of peripheral ETASTs to predict immunotherapy efficacy was demonstrated by monitoring changes in peripheral blood ETASTs in 40 NSCLC patients before and after immunotherapy. This approach offers a promising and efficient predictive biomarker for immunotherapy with high accuracy, specificity, accessibility, and minimal trauma (**Figure** **1**).

## 2 Results

### 2.1 Preparation and Characterization of TATAN Loaded with Whole‐Cell Tumor Antigens

Whole‐cell tumor antigens (including water‐soluble antigens and 8 M urea‐solubilized water‐insoluble antigens) were encapsulated as previously described in our studies.^[^ ^28^, ^29^, ^30^ ^]^(Figure S1a, Supporting Information). These NPs have an average size of ≈210 ± 30 nm, with a Zeta potential of ‐20 ± 2 mV and a PDI value of 0.163 ± 0.052 (Table S1, Supporting Information). The particle size was controlled within a range efficiently taken up by APCs.^[^ ^31^ ^]^ The loading capacities of all these NPs are ≈0.73 mg antigen /mg PLGA. Transmission electron microscopy (TEM) revealed that the NPs exhibited a regular spherical structure (Figure S1b,c, Supporting Information). Flow cytometry analysis showed that all three types of APCs, namely B cells (B220), mononuclear macrophages (F4/80), and DC (CD11c), could take up TATAN (Figure S1d,e, Supporting Information). Confocal microscopy results (Figure S1f, Supporting Information) revealed that while the red fluorescence signal of the nanoparticles partially overlapped with the green fluorescence signal of the cell lysosomes in some areas, there were also instances where the red fluorescence signal was observed in the cytoplasm, indicating the successful escape of most nanoparticles from the lysosomes into the cytoplasm.

**Figure 2:**
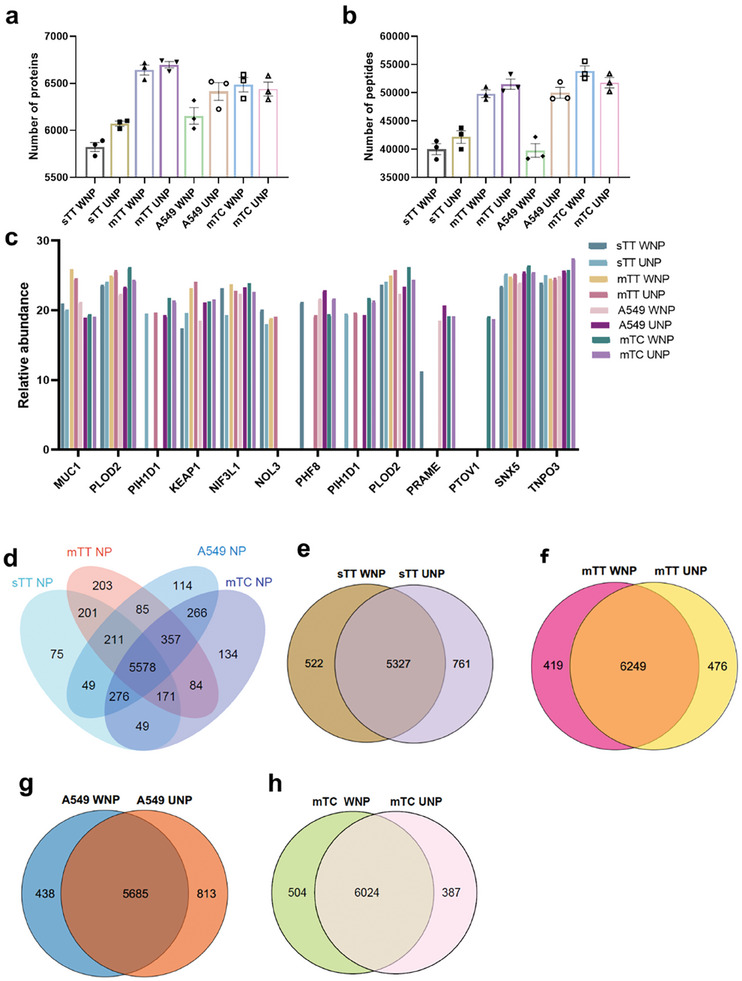
Proteomic mass spectrometry analysis of components in different TATANs. a) Protein species loaded in different TATANs. b) Peptide species loaded in different TATANs. c) Relative abundance of neoantigens loaded into different TATANs. d,e) In comparing protein species loaded in different NPs, overlap indicates sharing among multiple NPs. sTT WNP: NPs loaded with water‐soluble antigens from a single tumor tissue; sTT UNP: NPs loaded with water‐insoluble antigens from a single tumor tissue; mTT WNP: NPs loaded with mixed water‐soluble antigens from multiple allogeneic tumor tissues; mTT UNP: NPs loaded with mixed water‐insoluble antigens from multiple allogeneic tumor tissues; A549 WNP: NPs loaded with water‐soluble antigens from a single cell line (A549); A549 UNP: NPs loaded with water‐insoluble antigens from a single cell line (A549); mTC WNP: NPs loaded with water‐soluble antigens from a mixture of multiple tumor cell lines; mTC UNP: NPs loaded with water‐insoluble antigens from a mixture of multiple tumor cell lines (A549, H1299, H1650, PC9, H226, H520, SK‐MES‐1).

Proteomic analysis was conducted to identify the proteins and peptide antigens carried by different TATANs, revealing that each nanoparticle loaded ≈5800–6800 protein species and 40 000–51 000 peptide species under a stringent standard of FDR < 0.01 (**Figure** **2**a,b). Further analysis revealed that proteins carried by the nanoparticles included various neoantigens derived from mutations recorded in the Catalog of Somatic Mutations in Cancer, such as PlOD2, KEAP1, and PIH1D1. TATANs loaded with a mixture of multiple tumor tissues or cell lines carried more diverse antigen species than those loaded with single tumor tissues or cell lines (Figure 2d). In addition, the inclusion of water‐insoluble components significantly enriched the antigen species encapsulated by TATANs (Figure 2e–h). Furthermore, NPs loaded with a mixture of multiple allogeneic tumor tissues or multiple mixed cancer cell lines contained a greater and more diverse range of antigens than those loaded with single tumor cells or tissues (Figure 2e–h), demonstrating the feasibility of using multiple mixed tumor tissues or multiple mixed cancer cell lines instead of autologous tumor tissues for TATAN preparation.

### 2.2 TATAN Efficiently Activates ETASTs Both In Vivo and In Vitro, and The Level of ETASTs Correlates Positively with Treatment Efficacy

**Figure 3:**
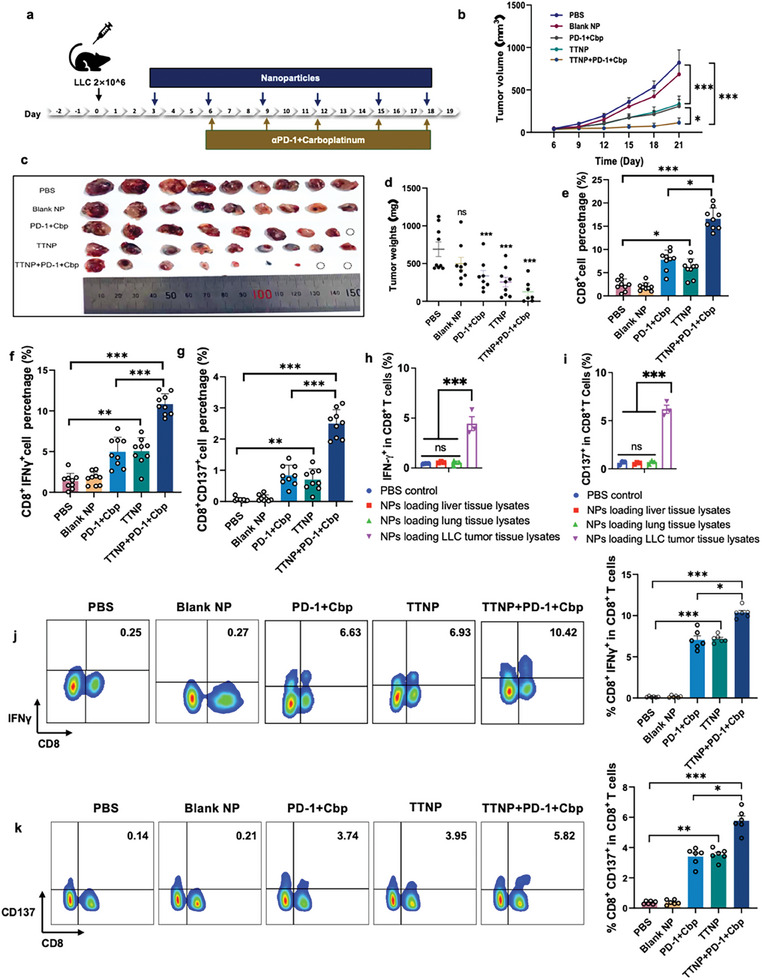
Increase of ETASTs is positively correlated with immunotherapy efficacy in a mouse model. a) Flowchart of tumor inoculation and drug administration. b) Tumor volume growth curves in mice. c) Photographs of isolated tumors in each group on day 21. d) Average weight of isolated tumors on day 21. e) Percentage of CD8^+^ T cells within the tumor as counted by immunofluorescence staining analysis. f) Percentage of CD8^+^IFNγ^+^T cells within the tumor, as counted by immunofluorescence staining analysis. g) Percentage of CD8^+^CD137^+^ T cells within the tumor as counted by immunofluorescence staining analysis. h) CD8^+^IFNγ^+^ tumor antigen‐specific T cells specificity analysis by comparing different tissue sources. i) CD8^+^CD137^+^ tumor antigen‐specific T cells specificity analysis by comparing different tissue sources. j) Representative flow cytometry result (left) and summary (right) of CD8^+^IFNγ^+^T cells in splenocytes. k) Representative flow cytometry result (left) and summary (right) of CD8^+^CD137^+^ T cells in splenocytes. NP, nanoparticles; Cbp, cisplatin; TTNP, nanoparticles loading whole tumor tissue lysates. Data are presented as mean ± SEM, and P‐values <0.05 were considered significant: ^*^P < 0.05, ^**^P < 0.01, ^***^P < 0.001.

The immune activation and cancer therapeutic efficacy of antigen‐loaded nanoparticles were evaluated in subcutaneous and orthotopic LLC lung cancer mouse models. Following tumor inoculation, either subcutaneously or in the lungs, the mice were injected with tumor tissue antigen‐loaded nanoparticles (TTNP) six times (**Figure** **3**a). Meanwhile, αPD‐1 antibody and carboplatin (Cbp) were administered in combination as a standard anti‐tumor regimen five times. In the subcutaneous mouse model and orthotopic mouse model, either TTNP or αPD‐1+Cbp effectively suppressed tumor growth (Figure 3b–d; Figure S2, Supporting Information), and combining TTNP with αPD‐1+Cbp can further improve the inhibition of tumor growth. No significant abnormalities were observed in the major organs of mice after treatment based on H&E staining, indicating minimal toxicity of the antigen NPs in mice (Figure S3a,b, Supporting Information).

**Figure 4:**
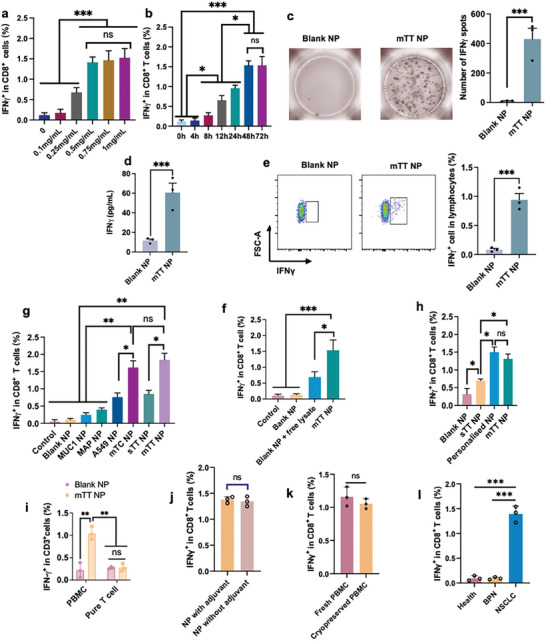
NPs loaded with whole tumor tissue antigens efficiently detect ETAST levels in the peripheral blood of NSCLC patients. a) Comparing different NP concentrations applied to activate and detect ETATS in blood. b) Comparing different co‐incubation times applied to activate and detect ETASTs in blood. c–e) Representative ETASTs detection results in ELISPOT, ELISA, and flow cytometry. f) NPs can more efficiently activate and detect ETASTs than free tumor lysates (patients = 3). g) Comparing different NPs loaded with different antigens to activate and detect ETASTs in blood (patients = 3). h) NPs loaded with a mixture of multiple allogeneic tumor tissue lysates showed similar detection effects with NPs loaded with autologous tumor tissue lysates (patients = 3). i) APC is needed to activate ETASTs (PBMC contains DC and B cells). j) No significant difference in ETASTs activated by mTT NP in fresh and frozen PBMC for two months. k) Comparing the amount of ETASTs detected by mTT NP in health people, people with benign pulmonary nodules, and NSCLC patients (patients = 3). l) Whether it carries an immune adjuvant or not does not affect NPs’ effectiveness in activating ETASTs (patients = 3). Blank NP, blank nanoparticles; MUC1 NP, NPS loaded with MUC1; MAP NP, NPs loaded with multiple neoantigen peptides; A549 NP, NPs loaded with whole cell lysates of A549; mTC NP, NPs loaded with a mixture of 7 tumor cell line lysates; sTT NP, NPs loaded with single tumor tissue lysates; mTT NP, NPs loaded with a mixture of 26 patient's whole tumor tissue lysates; Lysate, free whole lysate from 26 patient's lung cancer tumor tissue; Personalized NP, NPs loaded with personalized autologous whole tumor tissue lysate. The percentage of CD8^+^IFNγ^+^ T cells represents the percentage of ETASTs, BPN, and benign pulmonary nodules; data are presented as Mean ± SEM. P‐values <0.05 were considered significant: ^*^P < 0.05, ^**^P < 0.01, ^***^P < 0.001.

Flow cytometry analysis revealed a notable increase in the CD8^+^ T cell population, macrophages (F4/80), dendritic cells (CD11c), B cells (B220), and NK cells (CD49b) in the tumor tissue microenvironment of the TTNP group compared to those in the PBS and blank NP groups (**Figure** **4**a,b; Figure S4, Supporting Information). Furthermore, a synergistic effect was observed when combining TTNP with αPD‐1+Cbp. These results were verified by multiplex immunofluorescence (mIF) analysis (Figure 3e; Figure S4, Supporting Information). CD8^+^CD137^+^ T cells or CD8^+^IFN‐γ^+^ T cells within tumor tissues denote ETASTs, which are more indicative of immune activation features compared to CD8^+^ T cells alone.^[^ ^32^ ^]^ These studies revealed that TTNP significantly increased ETAST levels in the TME (Figure 3f,g; Figures S5 and S6, Supporting Information), and combining it with αPD‐1 further increased ETAST levels in the tumor. An increase in intra‐tumoral ETAST levels leads to improved antitumor therapeutic efficacy. T cell depletion studies revealed that CD8^+^ T cells function as the major killer cells, while CD4^+^ T cells also play a role in killing cancer cells (Figure S7, Supporting Information). These data reaffirm that TATAN activates ETASTs in vivo, correlating with improved therapeutic outcomes.

The specificity of NPs loaded with whole tumor antigens was verified by investigating NPs loaded with liver tissue lysates and NPs loaded with lung tissue lysates under the same conditions. The results demonstrated that NPs loaded with liver or lung tissue lysates did not activate tumor antigen‐specific T cells, thereby verifying the NPs’ specificity (Figure 3h,i).

Quantitative analysis of ETASTs in peripheral tissues from all mouse groups revealed that TATAN‐stimulated T cells capable of recognizing tumor antigens and activated ETASTs can secrete anti‐tumor cytokine IFN‐γ and express CD137. Thus, IFN‐γ^+^ T cells and CD137^+^ T cells (Figures S5 and S6, Supporting Information), particularly CD8^+^IFNγ^+^ T cells and CD8^+^CD137^+^ T cells (Figure 3j,k), are identified as ETASTs. Analysis of the correlation between the anti‐tumor effect and peripheral ETAST levels in each group indicated that better anti‐tumor efficacy resulted in higher levels of peripheral ETASTs, suggesting a positive correlation between the increase in peripheral ETASTs induced by TATAN and therapeutic efficacy.

### 2.3 TATAN Loaded with Whole‐Cell Antigens from Various Tumor Tissues Effectively Quantifies ETAST Levels in Patients with NSCLC Peripheral Blood

ETASTs in the peripheral blood of patients with NSCLC were activated by TATAN, followed by detecting activation markers IFN‐γ and CD137 for the quantitative analysis of ETASTs. Based on the screening studies, 0.5 mg mL^−1^ was selected as the co‐culturing concentration with PBMCs, and 48 h was determined to be the optimal in vitro co‐culturing time for activating ETASTs by TATAN (**Figure** **4**a,b; Figure S8a–c, Supporting Information).

Enzyme‐linked immunospot assays (ELISPOT), enzyme‐linked immunosorbent assays (ELISA), and flow cytometry are techniques commonly employed to quantify the number of T cells in an activated state. This study divided TATAN‐activated PBMC samples into three groups and subjected them to flow cytometry, ELISA, and ELISPOT to evaluate the activated T cells. Remarkably, the results from all three methods were consistent (Figure 4c–e), confirming that all three methods could be applied to measure the expression of activation‐state markers to quantify ETAST content. In this study, flow cytometry was used for subsequent investigations.

Furthermore, NPs loaded with whole tumor tissue lysates exhibited much better ETAST activation and detection effects than free tumor tissue lysates with blank NP (Figure 4f; Figure S8d, Supporting Information). In addition, the diversity of tumor antigens in TATAN was further compared by detecting ETASTs in the PBMCs of patients with NSCLC. The outcomes revealed that mTT NPs (loaded with tumor antigens from mixture of 26 allogeneic tumor tissues of patients with NSCLC) and mTC NPs (loaded with tumor antigens from mixture of 7 lung cancer cell lines) activated more CD8^+^IFNγ^+^ T cells compared to other NPs loaded with tumor antigens from single tumor tissue or single cell line or multiple neoantigen peptides (Figure 4g; Figure S8e, Supporting Information).

Importantly, mTT and mTC NPs achieved the same efficacy as personalized NPs loaded with autologous tumor tissues in activating and detecting ETASTs, whereas NPs loaded with single allogeneic tumor cell lysates did not achieve similar effects (Figure 4h; Figure S8f, Supporting Information). The fact that NPs can only activate antigen‐specific T cells in PBMCs (containing APCs such as B cells and DCs) but cannot activate pure T cells illustrates the necessity of including APCs in the co‐incubation system (Figure 4i)

In addition, at the applied NP concentration, we found that whether TATAN carried an immunoadjuvant did not affect the in vitro results of detecting ETASTs (Figure 4j). The content of TATAN‐activated ETASTs in PBMCs frozen in liquid nitrogen for two months was not significantly different from that in fresh PBMCs (Figure 4k), suggesting that cryopreserved PBMC samples can also be assayed for the content of ETASTs.

These data demonstrate that a mixture of multiple allogeneic tumor tissues and multiple tumor cell lines contains broad‐spectrum and diverse tumor antigens that sufficiently encompass the tumor antigens found in most patients with cancer. These findings confirm the feasibility of using a mixture of multiple allogeneic tumor tissues or multiple tumor cell lines as a substitute for autologous tumor tissue. Therefore, NPs loaded with a mixture of multiple allogeneic tumor tissue lysates or those loaded with a mixture of multiple tumor cell line lysates can be used as a universal TATAN to detect ETASTs in the peripheral blood of patients with cancer.

More importantly, the amount of ETASTs in the blood of patients with lung cancer, as detected by NPs loaded with a mixture of allogeneic tumor tissue lysates from 26 patients, was significantly higher than that in healthy individuals and individuals with benign pulmonary nodules (Figure 4l). These findings indicate that tumor tissues in patients with cancer can stimulate certain ETASTs in their bodies, which is a fundamental function of ICIs.

### 2.4 General Clinical Characteristics of NSCLC Patients Undergoing Chemoimmunotherapy

We enrolled 40 previously untreated patients with NSCLC undergoing this regimen to assess the potential of TATAN‐activated peripheral ETAST levels as biomarkers for predicting the efficacy of PD‐1 inhibitors combined with chemotherapy in NSCLC treatment. The median age of the enrolled patients was 63 years, with 25 (62.5%) patients aged > 65 years. Clinical efficacy was evaluated using RECIST 1.1 criteria at week 6 from the beginning of treatment, revealing that 14 (35.0%) patients achieved complete remission (CR) or partial remission (PR), 21 (52.5%) exhibited stable disease (SD), and 5 (12.5%) showed disease progression (PD), resulting in an objective response rate (ORR) of 35.0% and a disease control rate (DCR) of 87.5%. PD‐L1 expression was assessed in all patients, with 11 patients (27.5%) exhibiting expression levels below 1%, 18 patients (47.5%) showing expression levels between 1% and 49%, and 10 patients (25.0%) showing expression levels exceeding 50% (Tables S2 and S3, Supporting Information)

Blood tests such as neutrophil‐to‐lymphocyte ratio (NLR), C‐reactive protein (CRP) index, lactate dehydrogenase (LDH) levels, and platelet count, have been reported to predict immunotherapy efficacy;^[^ ^33^, ^34^, ^35^, ^36^, ^37^ ^]^ therefore, we also examined these biomarkers in this study and found no significant differences in platelet count (median 182.5 versus 178.5 10^9 /L), lactate dehydrogenase level (median 173.2 vs. 196.6 U L^−1^), and C‐reactive protein level (median 6.4 vs. 4.3 mg L^−1^) between patients in the responder (R) and non‐responder (NR) groups before treatment (Figure S9a–c, Supporting Information). However, the NLR was significantly lower in the R group than in the NR group (median 1.6 vs. 2.7) (Figure S9d, Supporting Information), and PD‐L1 expression was slightly higher in the R group than in the NR group (median, 25.0% vs. 9.5%) (Figure S9e, Supporting Information). Subsequently, we constructed receiver operating characteristic (ROC) curves for the NLR and PD‐L1 expression to evaluate their predictive ability for treatment responses. The area under the curve (AUC) for NLR was 0.776 (95% CI: 0.627–0.926) with an accuracy of 0.769, whereas the AUC for PD‐L1 expression was 0.713 (95% CI: 0.514–0.860) with an accuracy of 0.783 (95% CI: 0.514–0.860) (Figure S9f,g, Supporting Information). These results reveal that although NLR and PD‐L1 expression levels can predict immunotherapy response, their accuracy is limited.

### 2.5 Changes in the Levels of ETASTs in the Peripheral Blood of NSCLC Patients Treated with Chemoimmunotherapy

We obtained 147 blood samples from 40 patients with NSCLC who underwent chemotherapy before treatment and after the first and second courses. Of these, 139 PBMCs were successfully isolated for further analysis. Flow cytometric analysis was initially conducted on PBMC in selected patients to assess the effect of immunotherapy on peripheral blood T cells, as illustrated by the gating strategy in Figure S10a (Supporting Information). The results showed no significant differences in the total CD4^+^ T cell levels among the various groups at all treatment stages. However, the total CD8^+^ T cell levels were slightly higher in the response group (Group R) than in the non‐response group (Group NR) after two treatment cycles (Figure S10b,c, Supporting Information). Although a previous study highlighted a significant association between the baseline ratio of CD4^+^ to CD8^+^ T cells and treatment response,^[^ ^38^ ^]^ in this study, no significant difference in the CD4/CD8 ratio was observed between the different response groups at baseline (Figure S10d, Supporting Information). We examined the number of ETASTs activated by TATAN in the peripheral blood of patients with NSCLC after various treatment cycles to investigate the specificity of TATAN. The results showed that Patients in group R, who underwent two treatment cycles, exhibited a significant increase in ETAST levels after co‐incubation with TATAN (Figure S10e,f, Supporting Information).

### 2.6 The Increase in ETASTs in Peripheral Blood after Treatment was Positively Correlated with Therapeutic Efficacy

**Figure 5:**
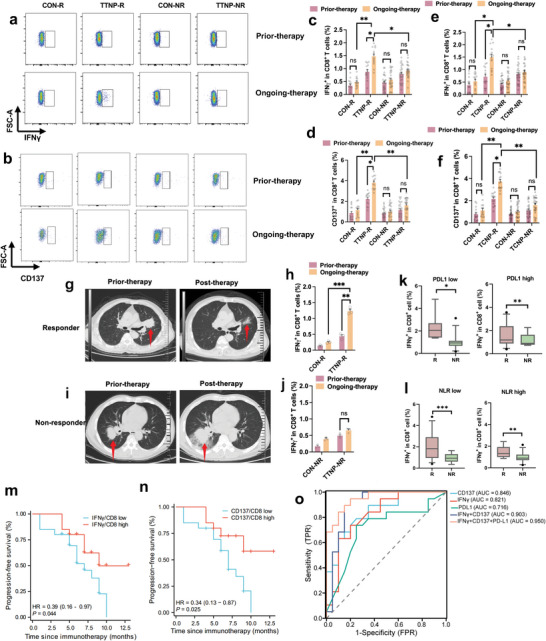
ETAST levels in peripheral blood positively correlate with immunotherapy efficacy. a) Representative flow cytometry analysis of CD8^+^IFN‐γ^+^ T cells activated by mTT NP in each group. b) Representative flow cytometry analysis of CD8^+^CD137^+^ T cells activated by mTT NP in each group. c) Analysis of CD8^+^IFN‐γ^+^ T cells activated by mTT NP responders and non‐responders (Each data point represents 1 patient, n = 40). d) Analysis of CD8^+^CD137^+^ T cells activated by mTT NP responders and non‐responders (Each data point represents 1 patient, n = 40). e) Analysis of CD8^+^IFN‐γ^+^ T cells activated by mTT NP responders and non‐responders (Each data point represents 1 patient, n = 40). f) Analysis of CD8^+^CD137^+^ T cells activated by mTT NP responders and non‐responders (Each data point represents 1 patient, n = 40). g,h), Representative chest CT images of responders and corresponding ETASTs test before therapy and after therapy. i,j), Representative chest CT images of responders and corresponding ETASTs test prior‐therapy and post‐therapy. k) Relationship between PD‐L1 expression and CD8^+^IFNγ^+^ T cell content. l) Relationship between NLR value and CD8^+^IFNγ^+^ T cell content. (m, n) Kaplan‐Meier survival curves demonstrating progression‐free survival of patients with different CD8^+^IFN‐γ^+^ and CD8^+^CD137^+^ T cell levels. o) ROC curves of ETAST predicting the effect of chemoimmunotherapy in patients with NSCLC, with IFN‐γ representing CD8^+^IFNγ^+^ T cells percentage and CD137 representing CD8^+^CD137^+^ T cells. Ongoing‐immunotherapy, two treatment cycles; R, treatment response group; NR, treatment non‐response group; CON, blank control group; TTNP, multi‐tumor tissue antigen nanoparticles; NLR, Neutrophil to lymphocyte ratio, neutrophil‐to‐lymphocyte ratio; HR, hazard ratio; statistical difference test using Log‐rank method; AUC, Area under the curve. Data are presented as Mean ± SEM. P‐values <0.05 were considered significant: ^*^ *P* < 0.05, ^**^ *P* < 0.01, ^***^ *P* < 0.001.

The association between peripheral blood ETAST levels and the efficacy of chemoimmunotherapy was explored by collecting PBMC samples from 40 patients before and after two treatment cycles. Subsequently, T cells expressing IFN‐γ^+^ and CD137^+^ after co‐incubation with TATAN (mTT NP and mTC NP) were evaluated using flow cytometry. Compared with ETASTs (CD8^+^IFNγ^+^ T cells or CD8^+^CD137^+^ T cells) in prior chemoimmunotherapy samples, ETASTs significantly increased in ongoing‐chemoimmunotherapy samples in the treatment‐responsive group (TTNP‐R) after co‐incubating PBMC with mTT NP. In samples of ongoing chemoimmunotherapy from the treatment‐response group, the percentage of CD8^+^IFN‐γ^+^ T cells and CD8^+^CD137^+^ T cells in total CD8^+^ T cells reached 1.81% ± 0.34% and 5.14% ± 1.72%, respectively (**Figure** **5**a–d). However, ETAST levels in ongoing chemoimmunotherapy samples from the non‐responder group (TTNP‐NR) did not notably increase after co‐incubating PBMC with TATAN. Meanwhile, the percentages of CD8^+^IFNγ^+^ T cells and CD8^+^CD137^+^ T cells in ongoing chemoimmunotherapy samples were the same as those in prior chemoimmunotherapy samples, both in responders and non‐responders, indicating that activating ETASTs with TATAN before flow cytometry analysis is indispensable. In addition, mTC NP and mTT NP showed similar ETAST detection (Figure 5e,f). Representative chest CT images of the responders and non‐responders before and after therapy are shown in Figure 5g,i). The corresponding ETAST levels are shown in Figure 5h,j, before and after post‐therapy, illustrating that increasing ETAST levels in the blood predicts good therapeutic efficacy. All these findings illustrate that after immunotherapy, the number of ETASTs recognizing tumor antigens increased significantly in the peripheral blood of responders but did not increase in the blood of non‐responders. Furthermore, this phenomenon was a general occurrence that can be observed across different pathological types (lung adenocarcinoma and lung squamous cell carcinoma), clinical stages (stages III and IV), and immune checkpoint inhibitors (pembrolizumab, tislelizumab, and sintilimab) as shown in Figure S11 (Supporting Information).

Patients were divided into two groups based on PD‐L1 expression, low and high, using the median PD‐L1 expression as the cutoff. Subsequently, ETAST levels in peripheral blood during ongoing chemoimmunotherapy were compared between responders and non‐responders within these groups. In both the low and high PD‐L1 expression groups, responders exhibited significantly higher ETAST levels than non‐responders (Figure 5k). Similar trends were observed in the low and high NLR groups (Figure 5l). These findings suggest that ETAST detection via TATAN activation could serve as an independent predictive marker of immunotherapy efficacy in patients with NSCLC, distinct from known markers such as PD‐L1 expression and pre‐treatment NLR.

### 2.7 ETAST Level in Peripheral Blood Predicts Prognosis in Chemoimmunotherapy for NSCLC

The final follow‐up for this study was conducted in February 2024, with a mean follow‐up duration of 9.5 months and an endpoint defined as either disease progression or patient death. The mean progression‐free survival (PFS) of all the enrolled patients was 6.3 months. Patients were stratified into low and high‐level groups based on the median levels of TATAN‐activated CD8^+^IFN‐γ^+^ T cells and CD8^+^CD137^+^ T cells in peripheral blood. Statistical analysis revealed that the mean PFS was 5.5 months for patients in the low‐level IFN‐γ/CD8 group and 7.3 months for those in the high‐level group. Similarly, for the low‐level CD137/CD8 group, the mean PFS was 5.3 months, whereas it was 7.4 months for the high‐level group. PFS survival curves for different ETAST content groups were plotted using the Kaplan‐Meier method, indicating (Figure 5m,n) a significant association between high levels of IFN‐γ/CD8 and CD137/CD8 and longer PFS, with hazard ratios (HRs) of 0.39 and 0.34, 95% confidence intervals of 0.16–0.97 and 0.13–0.87, and P‐values of 0.044 and 0.022, respectively. These findings suggest that ETAST levels in the peripheral blood could serve as important biomarkers for predicting the prognosis of patients undergoing chemotherapy for lung cancer. However, owing to the short follow‐up duration and limited occurrence of patient death, the relationship between ETAST and overall survival (OS) was not analyzed in this study.

### 2.8 ETAST in Peripheral Blood is a Predictive Biomarker of Chemoimmunotherapy Efficacy

The performance of ETAST levels in predicting the treatment response was assessed using receiver operating characteristic curve analysis. The AUC for CD8^+^IFNγ^+^ T cells and CD8^+^CD137^+^ T cells was 0.821 and 0.846, respectively (Figure 5o), demonstrating superior predictive efficacy compared to PD‐L1 expression (AUC = 0.716). Combining these two ETAST markers increased the AUC to 0.903, with a sensitivity of 0.913 and a specificity of 0.884. Combining three biomarkers — CD8^+^IFNγ^+^ T cells, CD8^+^CD137^+^ T cells, and PD‐L1 expression — further improved the AUC to 0.950, with sensitivity rising to 0.962 and specificity reaching 0.944. These findings affirm that peripheral blood ETAST levels have a significant predictive value for anticipating a response to chemoimmunotherapy after immunotherapy.

### 2.9 Analysis of Immune Cell Subsets in PBMC with and without TATAN Activation

**Figure 6:**
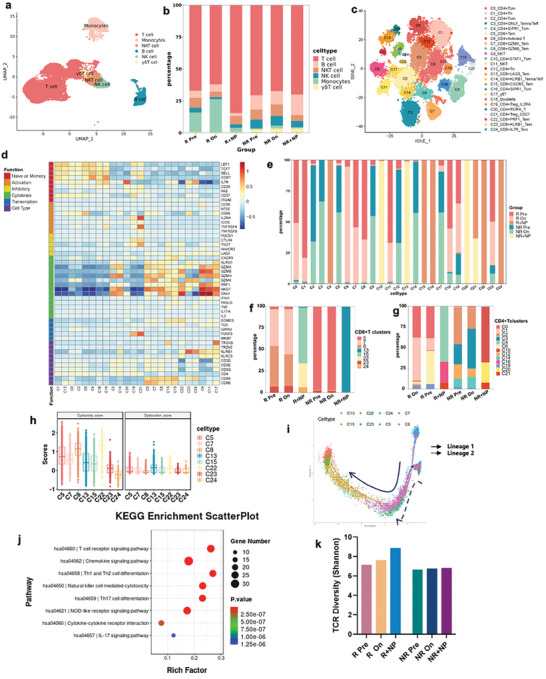
Analysis of immune cell subtypes in PBMCs of patients with NSCLC with and without TATAN activation. a) Clusters of subtypes in immune cells in PBMCs based on the expression of known marker genes. b) Percentage of each cell type in PBMC samples. c) T‐cell subpopulations were re‐clustered to obtain a total of 25 clusters of T cells. d) Heatmap showing the expression of selected genes in each cell subpopulation, representing naïve, memory, cytotoxicity, activation type, functionally depleted, and transitional T‐cell immune phenotypes. e) Percentage of each T‐cell subtype in all sample subpopulations. f) Different CD8^+^ T cell clusters in responders and non‐responders to immunotherapy. g) Different CD4^+^ T cell clusters in responders and non‐responders to immunotherapy. h) Cytotoxicity and functional depletion scores in CD8^+^ T cells. i) Mimetic temporal analysis of CD8^+^ T cells. j) KEGG enrichment analysis of C15 cell clusters for differential gene pathways. k) Diversity of T‐cell receptor (TCR) of CD8^+^ T cells (Shannon index) in responders and non‐responders to immunotherapy. T_N_, naïve T cells; T_CM_, central memory T cells; T_EM_, effector memory T cells; T_eff_, effector T cells; T_EMRA_, terminally differentiated effector memory T cells; T_reg_, regulatory T cells. Pre, pre‐immunotherapy; On, Ongoing‐immunotherapy; R, treatment response group; NR, treatment non‐response group; NP, nanoparticles loading mixed lysates of 26 allogeneic tumor tissues.

Single‐cell sequencing was conducted on six PBMC samples from two lung adenocarcinoma patients with different responses to chemoimmunotherapy to delve deeper into the influence of TATAN on immune cell activation in peripheral blood. These patients were closely matched in terms of age, gender, staging, PD‐L1 expression, and gene mutation status, with each patient providing three blood samples: pre‐treatment, ongoing‐treatment, and ongoing‐treatment blood activated by TATAN. Following quality control, 59428 cells were analyzed, with a median of 1290 genes detected per cell. Unsupervised clustering was performed to classify the cells from the six samples, and the cell types were identified based on characteristic molecular markers. The analysis revealed 22 distinct clusters encompassing all the cells (Figure S12a, Supporting Information). Clusters 0, 1, 2, 3, 4, 9, 10, 11, 14, and 17 were identified as T cells, characterized by the expression of genes such as CD3D, CD3E, and CD3G,. Clusters 7, 16, and 18, which expressed genes CD19, MS4A1, and CD79A, respectively, were classified as B cells. Clusters 5, 12, 15, and 19 were categorized as monocytes, expressing genes such as CD14, LYZ, and CD68. Cluster 13, expressing TRGV9 and TRGV2 genes, was identified as γδ T cells, while Cluster 6 was designated as NKT cells, Cluster 8 as NK cells, and Cluster 21, characterized by high expression of *PPBP* genes, was excluded from subsequent analysis (**Figure** **6**a; Figure S12b, Supporting Information).

The stacked histograms illustrated the percentage occupancy of each cell subset in the PBMC samples (Figure 6b). Before treatment, responders and non‐responders had similar T‐cell occupancy, and the total number of T‐cells did not change significantly after two treatment cycles. However, the total number of T cells significantly increased after co‐incubation with antigenic NPs. The total number of B cells decreased in responders (R+NP group) but not in non‐responders (NR+NP group). In addition, the proportion of monocytes in the peripheral blood of responders is significantly higher than that in non‐responders, while the number of γδ T cells and NK cells was significantly lower.

### 2.10 T Cell Subtypes in Peripheral Blood of NSCLC Patients Activated by TATAN

T cells are the primary immune cells responsible for killing cancer cells, and we re‐clustered 31931 previously identified T cells to obtain 25 clusters of T‐cell subtypes (Figure 6c). These clusters were annotated based on differentiation‐ and function‐related marker genes^[^ ^39^, ^40^ ^]^ (Figure 6c,d). Specifically, 8 clusters were annotated as CD8^+^ T cells (C5, C7, C8, C13, C15, C22, C23, and C24), while 13 clusters were identified as CD4^+^ T cells (C1, C12, C0, C2, C6, C4, C16, C10, C3, C14, C20, C21, and C19). Clusters C9 and C11 were annotated as NKT cells, C17 as γδ T cells, and C18 as adherent bipartite cells. These T‐cell subtypes were characterized based on genomic expression, representing various immunophenotypes, including naïve, memory, cytotoxic, activated, functionally depleted, and transitional (Figure 6d). Marker genes associated with naïve and memory traits, such as TCF7, CCR7, and SELL, were predominantly expressed in CD4^+^ naïve T cells (C1, C12). Cluster C6 cells expressed both naïve and memory trait genes and high levels of activation‐specific genes, like CD69. Clusters C3 and C14 represented CD4^+^ effector T cells with high expression of cytotoxic genes such as GZMA, GZMB, and GZMH. Clusters C5, C7, C8, C13, C15, and C22 were identified as CD8^+^ effector T cells. Functionally depleted signature genes, such as PDCD1, CTLA‐4, and TIGIT, were primarily expressed in CD4^+^ T_reg_ cells (C19 and C21).

Analysis of the percentage of each T‐cell subset before and ongoing chemoimmunotherapy revealed significant changes (Figure 6e–g). Specifically, the C6_CD4^+^Activated T‐cell subset showed a notable increase in treatment responders after two cycles of treatment, whereas non‐responders exhibited increases mainly in C3_CD4^+^GNLY_Temra/Teff and C17_γδ T‐cells. Memory and effector T cells significantly increased in responders’ peripheral blood after co‐incubation with TATAN, including C4_CD4^+^SIPR1_Tem, C16_CD4^+^SIPR1_Tcm, C15_CD8^+^CXCR3_Tem, and C22_CD8^+^PRF1^+^_Tem. In contrast, non‐responders experienced increased levels mainly in C3_CD4^+^GNLY_Temra/Teff and C17_γδ T cells after co‐incubation with TATAN. Notably, co‐incubation led to increased numbers of C10_CD4^+^STAT1_Tcm, C13_CD8^+^LAG3_Tem, and C20_CD4^+^RORA_T cells in the peripheral blood of non‐responders. Comparison of the gene expression profiles of the C15_CD8^+^CXCR3_Tem cluster and C22_CD8^+^PRF1^+^_Tem in responders and the C13_CD8^+^LAG3_Tem cluster in non‐responders revealed similarities in gene expression. However, the C13 cluster exhibited higher expression levels of the depletion‐related genes LAG3 and TIGIT, whereas the C15 cluster showed higher expression levels of the cytotoxic function‐related gene GZMK. The cytokine ligand genes CXCR3 and C22_CD8^+^ PRF1^+^_Tem showed higher cytotoxic function‐related GZMB and PRF1 expression levels. Functional genes and scores further validated this difference (Figure 6h); C15_CD8^+^CXCR3_Tem had similar cytotoxicity scores to C13_CD8^+^LAG3_Tem, and C22_CD8^+^ PRF1^+^_Tem had higher cytotoxicity scores than C15_CD8^+^CXCR3_Tem and C13_CD8^+^LAG3_Tem, whereas C13_CD8^+^LAG3_Tem had significantly higher functional depletion scores than C22_CD8^+^ PRF1^+^_Tem and C15_CD8^+^CXCR3_Tem.

Pseudo‐time analysis revealed that CD8^+^ T cells exhibited two main differentiation starting points, C5_CD8^+^Tem cells and C8_CD8^+^GZMB_Tem cells (Figure 6i), and then progressed along a trajectory that passed through C7_CD8^+^GZMK_Tem, C23_CD8^+^KLRB1_Tem, and C24_CD8^+^IL7R_Tem, and finally differentiated into C22_CD8^+^PRF1^+^_Tem and C15_CD8^+^CXCR3_Tem PRF1^+^ with C13_CD8^+^LAG3_Tem cells in the terminal differentiation state. This differentiation pathway correlated with the highest cellular functional exhaustion scores observed in the C22 and C13 clusters. This differentiation pathway correlated with the highest cellular functional exhaustion scores observed in the C22 and C13 clusters.

Subsequently, we analyzed the enrichment of differentially expressed genes in C15_CD8^+^CXCR3_Tem cells using the Kyoto Encyclopedia of Genes and Genomes (KEGG) database. The results revealed that the highly expressed genes in C15_CD8^+^CXCR3_Tem cells were enriched in pathways related to chemokine signaling, T cell receptor signaling, natural killer cell‐mediated cytotoxicity, and IL‐17 signaling (Figure 6j). Additionally, the diversity of T cell receptors (TCR) in responders to immune checkpoint inhibitors can be increased after immunotherapy; thus, we investigated TCR diversity in responders and non‐responders to immunotherapy. The TCR diversity of CD8^+^ T cells, obtained through single‐cell sequencing of TCR, was analyzed using the Shannon index, which demonstrated that immunotherapy increased TCR diversity in response to immunotherapy (Figure 6k). TATAN activation can further amplify the increase in TCR diversity between responders and non‐responders. This amplification suggests NP stimulation effectively activates CD8^+^ T cells to exert cytotoxic effects. In summary, our single‐cell sequencing results demonstrate that TATAN can reactivate CD8^+^ T cell subpopulations with high expression of cytotoxic genes in the peripheral blood of responders to chemoimmunotherapy, identifying this subpopulation as the activated ETAST cell population.

### 2.11 ETASTs Isolated from the Blood of Non‐Small Lung Cancer Patients can Efficiently Kill Autologous Cancer Cells In Vitro

CD3^+^CD137^+^ ETASTs isolated from the peripheral blood of two lungs of patients with cancer were further evaluated for their efficacy in killing autologous cancer cells. Lung cancer cells were successfully isolated and expanded from these two patients with NSCLC. The specificity was verified by evaluating the cytotoxic efficacy of ETAST isolated from the blood of patients with cancer against autologous cancer cells from tumor tissues; CD3^+^CD137^+^ ETASTs were isolated from the peripheral blood of two lungs of patients with cancer mTC NPs loaded with mixed lysates of seven lung cancer cell lines. These isolated CD137^+^ ETASTs were expanded in vitro for ≈2 weeks, and the CD137^+^ ETASTs were co‐incubated with autologous cancer cells isolated from the tumor tissue of the corresponding patient with cancer.

**Figure 7:**
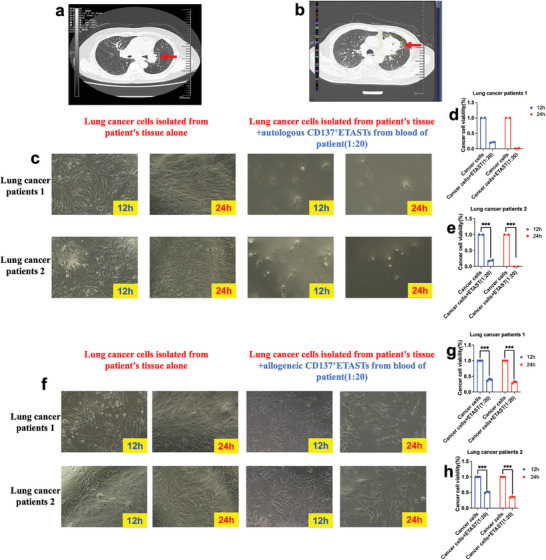
CD3^+^CD137^+^ ETAST isolated from blood of lung cancer patients can efficiently kill all autologous cancer cells from tumor tissue in vitro. a) CT images of non‐small lung cancer patient 1. b) CT images of patients with non‐small lung cancer 2. c) The images of isolated patients’ lung cancer cells after co‐incubating with autologous ETASTs (CD137^+^) isolated from the blood of corresponding patients with cancer. d) Cell viability of isolated lung cancer cells (from patient 1) after co‐incubating with autologous ETASTs (CD137^+^) isolated from the blood of patients (1) with cancer. e) Cell viability of isolated lung cancer cells (from patient 2) after co‐incubating with autologous ETASTs (CD137^+^) from the blood of patient (2) with cancer. f) The images of isolated patient's lung cancer cells after co‐incubating with allogeneic ETASTs (CD137^+^) isolated from the blood of another patient with cancer g) Cell viability of isolated lung cancer cells (from patient 1) after co‐incubating with allogeneic ETASTs isolated from the blood of patient (2) with cancer. h) Cell viability of isolated lung cancer cells (from patient 2) after co‐incubating with allogeneic ETASTs from the blood of patient (1) with cancer.

The CT images of the tumor tissues of these two lungs of patients with cancer are shown in **Figure** **7**a,b to illustrate tumor tissues in these patients. ETASTs and autologous lung cancer cells were co‐incubated for 12 h or 24 h before taking images or performing CCK8 analysis. The results demonstrated that ETASTs isolated from the PBMCs of patients with cancer could efficiently kill all autologous lung cancer cells in vitro (Figure 7c–e). In contrast, ETASTs isolated from the PBMCs of patients with cancer could only kill some allogeneic lung cancer cells from different patients with cancer in vitro (Figure 7f–h). These results further confirmed that TATAN‐activated ETASTs are tumor antigen‐specific T cells and cytotoxic effector T cells.

## 3 Discussions and Conclusions

Cancer involves competition between tumor cells and the immune system; current biomarkers focus on investigating tumor cell properties. The ability to quantify tumor‐specific immune responses is lacking; this study provides a method for accurately measuring tumor‐specific immune responses in patients with cancer. Thus, information on tumor cells and tumor‐specific immune responses can be provided to doctors and patients to select a better therapeutic strategy.

PD‐1 and PD‐L1 inhibitors significantly improve the prognosis of patients with advanced NSCLC.^[^ ^41^ ^]^ However, a considerable number of patients do not derive any clinical benefit, and reliable predictive markers for treatment responses are currently lacking. Tumor‐specific T cells in the tumor microenvironment have been extensively studied and directly implicated in cancer immune responses, strongly correlating with the efficacy of cancer immunotherapy.^[^ ^42^ ^]^ However, obtaining sufficient tumor tissue samples to detect tumor‐specific T cells in the tumor microenvironment can be challenging. Tumor‐specific T cells in tumor tissues are derived from the infiltration of ETASTs in peripheral blood; there is a good correlation between the immune cell profiles in peripheral blood and the tumor microenvironment.^[^ ^43^ ^]^ Given the safety and accessibility of peripheral blood testing, detecting TASTs in the peripheral blood to predict immunotherapy efficacy presents clear advantages.^[^ ^44^ ^]^ In this study, we developed nanoparticles loaded with whole‐cell tumor antigens capable of activating pre‐existing ETASTs through APCs in the peripheral blood. The content of these T cells can be quantitatively analyzed using characteristic markers of activated TASTs, which offer high specificity, minimal invasiveness, and high accessibility. By monitoring the changes in ETASTs content in the peripheral blood before and after immunotherapy in 40 patients with NSCLC, we demonstrated the feasibility of using this NP activation assay to predict immunotherapy efficacy, providing a highly specific, accessible, and accurate biomarker for predicting immunotherapy efficacy in lung cancer.

The detection of predictive markers for immunotherapy responses, such as PD‐L1 expression, TMB, and mIHC, typically requires sufficient tumor tissue, which is unsuitable for patients who lack access to such tissue. Alternative strategies utilizing immune cell‐specific signatures in the peripheral blood to gauge immunotherapy responses have been extensively explored. Parameters such as the neutrophil‐to‐lymphocyte ratio (NLR), CD4^+^/CD8^+^ T‐cell ratio, C‐reactive protein, and lactate dehydrogenase levels have been correlated with immunotherapy response;^[^ ^33^, ^34^, ^35^, ^36^, ^37^ ^]^ however, enhancing the predictive accuracy of these biomarkers remains a challenge. Among these, peripheral blood ETASTs show considerable promise. For instance, CD8^+^ PD‐1^+^ T cells, CD8^+^ PD‐1^+^ TIGIT^+^ T cells, and CD137^+^ T cells in the peripheral blood have effectively predicted immunotherapy response.^[^ ^45^, ^46^, ^47^ ^]^ However, these methods are insufficient, especially prospectively, for predicting the long‐term prognosis of patients with cancer.

CD8^+^IFNγ^+^ T cells are pivotal in the tumor immune response, engaging in activities such as direct tumor cell elimination and modulation of immune cell activation.^[^ ^47^, ^48^ ^]^ They are recognized as important indicators of immunotherapy response. However, IFNγ cannot be expressed unless antigen‐specific T cells encounter corresponding antigens, which makes it a highly specific marker for structural recognition and cytotoxic function. CD137 (4‐1BB) is a costimulatory receptor in the tumor necrosis factor receptor superfamily (TNFRSF) considered a hallmark of ETAST.^[^ ^49^ ^]^ Circulating CD137^+^ T cells in peripheral blood serve as biomarkers for predicting early recurrence and clinical remission in patients with metastatic renal cancer; elevated CD8^+^CD137^+^ T cell levels correlate with longer disease remission durations.^[^ ^50^ ^]^ However, CD8^+^IFN‐γ^+^ T cells and CD8^+^CD137^+^ T cells are rare without encountering tumor antigens because they are activation biomarkers and cytotoxic biomarkers. Our study detected IFN‐γ and CD137 as markers of ETASTs after encountering tumor antigens, making the detection more specific and accurate. As expected, a significant increase in CD8^+^IFN‐γ^+^ and CD8^+^CD137^+^ T cells in the peripheral blood of immunotherapy responders following co‐incubation with TATAN was observed. Notably, ETASTs in responder peripheral blood were predominantly “precursor‐depleted T cells,” which regain functionality after two cycles (42 days) of chemoimmunotherapy.^[^ ^51^, ^52^ ^]^ They rapidly activate upon re‐exposure to tumor antigens presented by APCs. Conversely, ETASTs in non‐responder peripheral blood are more likely to be terminally depleted T cells incapable of rejuvenation.^[^ ^53^ ^]^ Single‐cell sequencing revealed a notably higher depletion score of ETAST cells in non‐responder peripheral blood than in responders.

This study utilized nanoparticles loaded with whole‐cell tumor antigens to identify ETASTs in peripheral blood efficiently. In vitro T‐cell activation using antigen nanoparticles simulates the indirect activation process via autologous APCs, resembling the activation of pre‐existing TASTs in the periphery through APCs after the phagocytosis of in vivo antigens. Consequently, it exhibited high specificity. After two cycles of PD‐1 inhibitor treatment, some dysfunctional TASTs in the peripheral blood of responders regained their functionality and transitioned into ETASTs, which were activated upon encountering recognizable tumor antigens presented by APCs in *vitro*, as evidenced by the expression of activation state markers. Conversely, the majority of anergic tumor antigen‐specific T cells in the peripheral blood of non‐responders failed to recover their function, resulting in a minimal increase in pre‐existing ETASTs post‐treatment.^[^ ^54^ ^]^

A study comparing the predictive efficacy of various PD‐1/PD‐L1 treatment response markers, including mIHC, PD‐L1 expression, TMB, and gene expression profiling (GEP),^[^ ^55^ ^]^ revealed AUC values of 0.79 for mIHC, 0.65 for PD‐L1 expression, 0.65 for GEP, and 0.69 for TMB. In contrast, the AUC values for TATAN‐activated CD8^+^IFNγ^+^ T cells and TATAN‐activated CD8^+^CD137^+^ T cells in predicting immunotherapeutic response in our study were 0.821 and 0.846, respectively, and the AUC values for combining TATAN‐activated CD8^+^IFNγ^+^ T cells with TATAN‐activated CD8^+^CD137^+^ T cells is 0.90. These values surpassed those reported in the literature for mIHC, PD‐L1 expression, TMB, and GEP,^[^ ^9^, ^56^, ^57^ ^]^ suggesting that the predictive capacity of ETASTs for immunotherapy response exceeds that of existing assays, notwithstanding the requirement for tumor tissue in this approach. One potential explanation is that most of these assays rely solely on pre‐treatment tissue samples, whereas the tumor microenvironment dynamically changes throughout the treatment process. Conversely, acquiring multiple peripheral blood samples is better suited for monitoring treatment‐induced alterations.

This study employed single‐cell sequencing to examine changes in T cell subsets in PBMC before and after immunotherapy, with and without co‐incubation with antigen nanoparticles. Responders exhibited a notable increase in activated CD8^+^ T cells in peripheral blood after 42 days from the beginning of treatment. In contrast, non‐responders showed elevated levels of CD4^+^ effector T cells and γδ T cells expressing genes like GNLY in peripheral blood, indicating significant variance in the peripheral immune milieu among patients with differing immune responses.^[^ ^58^ ^]^ The expanded T cell subpopulations after co‐incubation with antigen nanoparticles for responders and non‐responders were C22_CD8^+^PRF1+_Tem, C15_CD8^+^CXCR3_Tem, and C13_CD8^+^LAG3_Tem. C22_CD8^+^PRF1+_Tem and C15_CD8^+^CXCR3_Tem tend to be functionally cytotoxic, whereas C13_CD8^+^LAG3_Tem tends to be exhausted and anergic.^[^ ^59^, ^60^ ^]^ Cluster 13 had high expression of LAG3 and TIGIT, whereas clusters 15 and 22 had high CXCR3/CCL5, PRF1, and GZMB expression. A study noted that immunotherapy‐induced CD8^+^ T cell populations exhibit active chemokine signaling (CXCR3/CCL5), lower levels of co‐suppressor receptors, higher cytotoxic gene expression, and upregulation of CXCR3 in neoantigen‐specific T cells.^[^ ^61^ ^]^ PRF1 and GZMB are cAs co‐suppressor receptors, along with LAG3 and TIGIT, in activated CD4^+^ and CD8^+^ T cells.^[^ ^62^ ^]^ Disparities in gene expression among C22_CD8^+^PRF1+_Tem, C15_CD8^+^CXCR3_Tem, and C13_CD8^+^LAG3_Tem offer insights into the distinct outcomes observed among different responders in peripheral blood. The stable frequency of high‐abundance clones of TCR diversity is closely associated with patients’ overall survival, and increasing TCR diversity in the blood could be related to the efficacy of immune checkpoint inhibitor (ICI) therapy.^[^ ^63^ ^]^ Our study also revealed a significant increase in TCR diversity among CD8^+^ T cells in responders compared to non‐responders, which may account for part of the underlying immunological mechanisms of increased ETASTs in responders. These single‐cell sequencing studies confirmed that more tumor‐specific T cells exist in patients’ blood and that responders and non‐responders have different features upon re‐encountering pan‐clone tumor antigens.

This detection is structurally and functionally specific by measuring the number of ETASTs activated by tumor antigens. In addition, the biggest challenge in accurately detecting all ETASTs is their high diversity and MHC (HLA) restriction, which were addressed by the method presented in this study using whole‐tumor antigen‐loaded NPs and APC‐mediated indirect activation of T cells. Pan‐clone ETASTs in the blood can be detected because the tumor antigens recognized by these ETASTs are loaded into the NPs (TATAN). Thus, the method presented in this study can detect more accurate, diverse, and comprehensive ETASTs in the peripheral blood of patients with cancer. Accurate measurement and monitoring of pan‐clone ETASTs in the blood of patients with cancer is important with the help of whole‐ETAST activation‐based detection. This study and a clinical trial demonstrated that the change in the amount of whole ETASTs in peripheral blood is positively correlated with therapeutic efficacy.

In summary, this study provides a highly accurate, highly specific, noninvasive, and facile biomarker for predicting immunotherapy efficacy in patients with lung cancer and potentially other cancers. Thus, more comprehensive information can be provided to doctors and patients to select better therapeutic strategies. Additionally, this method can be applied to evaluate the therapeutic efficacy of other cancer treatment methods, such as radiotherapy, photothermal therapy, oncolytic viruses, and nanomedicine‐based therapies.

## 4 Experimental Section

### Reagents

PLGA with MW 7000–17 000 (719 897, acid terminated, 50:50), PVA (360 627, MW: 9000–10 000 Da) were purchased from Sigma–Aldrich; poly (I: C) (vac‐pic) was purchased from InvivoGen; LLC, A549, H1299, H1650, PC9, H226, H520, SK‐MES‐1 cell lines were purchased from the Shanghai Cell Bank, Chinese Academy of Sciences; Dulbecco's modified eagle medium (DMEM)/High Glucose, RPMI 1640, and fetal cell lines were purchased from Sigma–Aldrich; Poly (I: C) (vac‐pic) was purchased from InvivoGen. RPMI 1640 and fetal bovine serum (FBS) were purchased from HyClone, and AIM V serum‐free medium was purchased from Thermo Fisher Scientific. MUC1, TRPAPGSTAPPAHGVTSAPDTRPAPGSTAP, CEA, YLSGANLNL, MAGE‐A3, and FLWGPRALV were purchased from Kingsley Bioscience, Inc. Collagenase/hyaluronidase and bovine serum were purchased from HyClone, and AIM V serum‐free medium was purchased from Thermo Fisher Scientific. Hyaluronidase and bovine pancreatic DNase were obtained from Stem Cell Technologies, and PD‐1 blocking antibody (BE0146, clone: RMP1‐14), anti‐CD8 antibody (BE0061, clone: 2.43) and anti‐CD4 antibody (Anti‐CD8 antibody (BE0061, clone: 2.43), and anti‐CD4 antibody (BE0003‐1, clone: GK1.5) were purchased from BioXcell. Detection nanoparticles ESMDT‐LLC‐1 and ESHDT‐HLC‐1 are provided by Suzhou Ersheng Biopharmaceutical Co., Ltd. All the antibodies used for flow cytometry were purchased from BioLegend. The Pan T Cell Lysolation Kit and Pan B Cell Lysolation Kit were purchased from Metheni Biotech, Germany, and the Fcioll reagent was purchased from Beijing Dako Bio Co. Fcioll reagent from Beijing Dako Bio Co. The carboplasts were purchased from Beijing Soleberg Technology Co.

### Ethics of Experimental Animals

Animal procedures were approved and monitored by the Animal Care and Use Committee of Soochow University (IACUC No. 202306A0667). The mice were housed in a specific pathogen‐free (SPF) animal room at the School of Pharmacy, Soochow University, with a constant temperature of 22 ± 1 °C, relative humidity of 50 ± 10%, and a 12 h artificial light cycle, and automatic ventilation, adhering to the guidelines outlined in the Guide for the Care and Use of Laboratory Animals.

### Ethics of Human Studies

This study was a prospective, non‐interventional investigation registered prospectively in February 2022 on Clinical Trials (https://classic.clinicaltrials.gov/) with registration number NCT05789498. This study was approved by the Ethics Committee of the First Affiliated Hospital of Soochow University (approval number 2 022 181). All experiments adhered to the principles of the Declaration of Helsinki, and all patients provided informed consent.

### Preparation of antigen nanoparticles loaded with water‐soluble cell components

The methods to prepare whole cancer cell line lysates (including both water‐soluble components and 8 M urea‐solubilized water‐insoluble components) and whole tumor tissues lysates (including both water‐soluble components and 8 M urea solubilized water‐insoluble components) were the same as it was previously reported. This study used a double‐emulsion method to prepare antigen nanoparticles using LLC cell lysates, peptides, human tumor cell lysates, and tumor tissue lysates from patients with lung cancer as antigens. The procedure was as follows: First, 300 µL of the water‐soluble fraction dissolved in endotoxin‐free water (80 mg mL^−1^, with 2 mg mL^−1^ Poly (I:C) added for in vivo treatment) was added to 1 mL of dichloromethane solution (containing 100 mg mL^−1^ PLGA) and sonicated for 1 min. Then (2.5 mL of PVA solution at a concentration of 20 mg mL^−1^ was added, followed by 45 s of sonication. The mixture was then dripped into a 50 mL solution containing 5 mg mL^−1^ of PVA and stirred at room temperature for 4 h to facilitate nanoparticle solidification. Finally, the supernatant was removed by centrifugation at 13 680 × g and the precipitate was resuspended in 10 mL of 4% trehalose solution. After freeze‐drying for 48 h, the nanoparticles were stored at −20 °C for future use. Throughout the preparation process, strict endotoxin‐free procedures were performed to ensure the quality of the NP vaccine. The antigen sources of the NPs used in this study were MUC1 NP, which are NPs loaded with a single peptide MUC1, a common tumor‐associated antigen. MAP NP: NPs loaded with three tumor‐associated antigens: MUC1, MAGE‐A3, and CEA. A549 NP: NPs loaded with whole‐cell antigens from lung cancer cells A549. mTC NP: NPsloaded with various lung cancer cell lines antigens (A549, H1299, H1650, PC9, H226, H520, SK‐MES‐1). sTT NP: NPs loaded with individual tumor tissue antigens from patients with NSCLC. mTT NP: NPs loaded with a mixture of tumor tissue antigens from multiple patients with NSCLC. These NPs were characterized according to the methods described in the previous publications.

### Preparation of Antigenic Nanoparticles Loaded with Water‐Insoluble Cellular Components

80 mg mL^−1^ of the water‐insoluble component was dissolved in 300 µL of 8 M urea, followed by adding 1 mL of 100 mg mL^−1^ PLGA in dichloromethane solution and sonication for 1 min. Subsequent steps were the same as those used to prepare antigenic Np loaded with water‐soluble cellular components. These NPs were characterized according to the methods described in the previous publications.

### Preparation of Antigen NPs Samples for Mass Spectrometry Analysis

Antigen NPs were dissolved in ultrapure water and mixed with four times the volume of acetonitrile to disrupt their structures. Subsequently, the mixture was left overnight at room temperature to evaporate acetonitrile, followed by vacuum drying of the remaining solution. The dried samples were collected for subsequent experiments related to mass spectrometry analysis. Each sample was supplemented with an appropriate amount of urea buffer (containing 8 M urea, 10 mM HEPES, 150 mM sodium chloride, pH = 8), transferred to EP tubes, and incubated at 37 °C for 1 h. The samples were then sonicated for 2 min, followed by centrifugation at 16 000 g for 20 min at 4 °C. The supernatant was collected for protein quantification using the BCA method. All protein samples underwent FASP digestion in the following specific steps: 1 M DTT was added to each sample to achieve a final concentration of 100 mM, followed by boiling water treatment for 5 min and cooling to room temperature. Subsequently, 200 µL UA buffer was added, mixed well, and transferred to 10 kDa ultrafiltration centrifuge tubes. Centrifugation was performed at 12 000 × g for 15 min. This step was repeated by adding UA buffer, centrifuging, and discarding the filtrate. IAA solution (50 mM IAA in UA) was added and incubated at room temperature in the dark for 30 min, followed by centrifugation for 10 min. The process of adding the UA buffer and centrifugation was repeated twice. NH_4_HCO_3_ buffer was then added, followed by two centrifugation cycles. Subsequently, Trypsin buffer (containing 6 µg Trypsin in 40 µL NH_4_HCO_3_ buffer) was added, followed by shaking for 1 min and incubation at 37 °C for 16–18 h. After changing the collection tubes, the filtrate was collected by centrifugation and supplemented with a 0.1% TFA solution. The peptide segments were desalted using a C18 Cartridge and freeze‐dried. The freeze‐dried peptide segments were resolubilized in 0.1% TFA, and their concentrations were determined and prepared for LC‐MS analysis. Mass spectrometry proteomic data were deposited in the ProteomeXchange Consortium (https://proteomecentral.proteomexchange.org) via the iProX partner repository with the dataset identifier PXD052576.

### Therapeutic Efficacy Studies on Tumor‐Bearing Mice

All mice used to establish the tumor mouse model were 6–8 weeks old C57BL/6J mice obtained from Shanghai Jihui Laboratory Animal Co. The subcutaneous tumor model was established by injecting 2 × 10^6^ LLC cells into the right hind legs of mice of appropriate age. The day of tumor cell inoculation was designated as day 0, and the tumor volume was measured every three days thereafter. Tumor volume was calculated using the formula: V (tumor volume) = 0.52 × a × b^2^, where a and b represent the long and short diameters of the tumors measured with Vernier calipers, respectively. The orthotopic tumor model was established by exposing the thoracic cavity of anesthetized mice through chest skin clipping, followed by the injection of 2 × 10^6^ LLC‐luc cells mixed with Matrigel into the lung apices. In situ, the bioluminescence of the tumors was examined using a near‐infrared small‐animal live imager to confirm successful inoculation. Tumor size was monitored using a small‐animal live imager based on fluorescence intensity on days 3, 8, 13, and 18. Mouse body weights were recorded every 3 days starting from day 0. Body weight and tumor volume were monitored, and mice were euthanized if their body weight decreased by more than 10% compared to the initial value or if the tumor volume exceeded 2000 mm^3^.

A subcutaneous LLC lung cancer model was established by inoculating LLC cells on day 0. The TTNP group received subcutaneous injections of 200 µL LLC tumor tissue antigen nanoparticles (containing 2 mg PLGA) on days 4, 7, 10, 13, 16, and 19, respectively. The PD‐1+Cbp treatment group was intraperitoneally injected with αPD‐1 antibody (10 mg kg^−1^) and carboplatin (20 mg kg^−1^) on days 7, 10, 13, 16, and 19. The TTNP+PD‐1+Cbp group received simultaneous subcutaneous injections of LLC tumor tissue antigen nanoparticles and intraperitoneal injections of αPD‐1 antibody and carboplatin. The Blank NP group received simultaneous injections of equal amounts of blank nanoparticles and tumor cell lysate components at different sites. The PBS control group received subcutaneous injections of equal volumes of PBS simultaneously. The treatment protocol for the in situ LLC lung cancer model was the same as that for the subcutaneous model.

### CD4^+^/CD8^+^ T Cell Depletion Experiment

A mouse model of subcutaneous LLC was used in this study. Two days before tumor inoculation, on the day of tumor inoculation, and every 4 days thereafter, mice received anti‐CD8 or anti‐CD4 antibodies to induce the depletion of CD8^+^ and CD4^+^ T cells, respectively. Tumor inoculation and treatment methods were consistent with those used in the TTNP^+^PD‐1^+^carboplatin treatment group. Each CD4^+^ T cell depletion group and CD8^+^ T cell depletion group comprised eight mice.

### Analysis of Tumor‐Infiltrating T Cell Content

The tumors were excised from the mice and minced into 1 –2 mm^3^ pieces in a culture medium. After mincing, the tissue fragments were incubated in a serum‐free DMEM medium containing collagenase IV and DNase‐I at 37 °C for 30 min to digest. The undissolved tissue was filtered through a mesh sieve, and the cell suspension was collected for centrifugation. Red blood cell lysis buffer was then added, followed by a 5 min incubation for erythrocyte lysis. After terminating the lysis reaction, the suspension was centrifuged again to remove the supernatant. The cells were transferred to round‐bottom centrifuge tubes, washed with PBS containing FBS, and centrifuged to remove the supernatant. Before surface staining, the cells were incubated with an Fc‐blocking reagent for 5 min and then stained with antibodies against mouse CD11c, CD49b, B220, F4/80, CD3, CD8, CD4, and CD137. The cells were fixed, permeabilized, and then stained with IFNγ for intracellular antibody staining.

### Patients and Samples

This study included untreated NSCLC patients who received a PD‐1 inhibitor combined with platinum‐containing chemotherapy at the First Affiliated Hospital of Soochow University from December 2022 to June 2023. All patients were diagnosed based on tumor tissue pathology results. General clinical data were collected, including patient name, age, gender, pathological classification, tumor staging, smoking history, ECOG‐PS score, metastasis status, treatment drugs, PD‐L1 expression, objective evaluation results of clinical efficacy, and follow‐up information. In addition, partial laboratory test results were collected before the first immunotherapy session, including neutrophil count, lymphocyte count, CD4^+^ T cell count, CD8^+^ T cell count, platelet count, serum lactate dehydrogenase (LDH) level, and C‐reactive protein level. The neutrophil‐to‐lymphocyte ratio (NLR) was defined as the ratio of absolute neutrophils to absolute lymphocytes. Patients were enrolled in 21‐day treatment cycles, with enhanced neck, chest, and abdomen scans conducted every two cycles to assess efficacy. PD‐L1 expression was evaluated using the Tumor Proportion Score (TPS), calculated as TPS = P (number of PD‐L1 membrane‐stained positive tumor cells/total tumor cells) ×100. TPS ≥ 50% indicated strong expression, 1% ≤ TPS ≤ 49% indicated expression, and TPS < 1% indicated no expression.

Inclusion criteria: 1) Patients with pathologically confirmed non‐small cell lung cancer receiving PD‐1 inhibitors combined with chemotherapy. 2) Age ≤ 18 years and ≤80 years. 3) ECOG PS performance status score of 0 or 1. 4) Adequate organ and bone marrow function. 5) Expected survival time ≥12 weeks. 6) Voluntary participation and signing of informed consent form.

Exclusion criteria: 1) Lack of assessable lesions. 2) Presence of tumor emergencies requiring immediate treatment. 3) Poor vascular condition. 4) Abnormal coagulation function or receiving thrombolytic or anticoagulant therapy. 5) Presence of blood‐borne infectious diseases such as HBV. 6) Presence of mental disorders or severe psychological illnesses. 7) Difficulty in communication or long‐term follow‐up. 8) Other conditions were not suitable for inclusion in this study.

### Evaluation of the Clinical Efficacy of Chemoimmunotherapy

The clinical efficacy evaluation was based on the Response Evaluation Criteria in Solid Tumors version 1.1 (RECIST 1.1). The maximum diameters of the target lesions were measured based on imaging examinations. The efficacy assessment criteria included Complete Response (CR), the disappearance of all target lesions; Partial Response (PR), at least a 30% reduction in the sum of the longest diameters of target lesions compared to baseline; Progressive Disease (PD), at least 20% increase in the sum of the longest diameters of lesions or the appearance of new lesions; and Stable Disease (SD), a reduction in the sum of the longest diameters of baseline lesions without reaching PR or an increase without reaching PD. Responders (R) were defined as individuals with tumor disappearance, tumor volume reduction, or stable volume for more than six months, whereas non‐responders (NR) were defined as individuals with tumor growth or clinical benefits lasting less than six months. The Objective Response Rate (ORR) was defined as the percentage of patients achieving CR and PR after treatment, whereas the Disease Control Rate (DCR) was defined as the percentage of patients achieving CR, PR, and SD among all patients.

### Isolation and Culture of PBMCs

Fresh whole blood was collected in sodium heparin anticoagulant tubes and diluted with equal PBS. Ficoll separation medium was added to sterile centrifuge tubes in an equal volume to whole blood, and the diluted blood sample was carefully layered on top of the separation medium. Centrifugation was conducted at room temperature at 800 g for 20 min with slow acceleration. After centrifugation, the peripheral blood mononuclear cell (PBMC) layer (white membrane layer) was aspirated and transferred to a 15 mL centrifuge tube. The cells were washed by adding 10 mL of PBS to the centrifuge tube, centrifuged at 3000 g for 5 min at room temperature, and the supernatant was discarded. The washing step was repeated 1–2 times. After cell counting, some cells were cryopreserved, whereas the other portion was resuspended in AIM V for further experimentation.

### Detection of ETASTs by Flow Cytometry

The thawed or freshly isolated PBMCs were resuspended and co‐incubated with antigen NPs (ESHDT‐HLC‐1) in a 96‐well plate cultured in a cell culture incubator at 37 °C for 48 h. Four hours before the end of the incubation period, BFA was added to each well. After 48 h of incubation, the PBMCs were collected from the incubator; detached adherent cells were pipetted, transferred to centrifuge tubes, and centrifuged at 1500 rpm for 5 min. The cells were resuspended in 1 mL PBS to obtain single cells kept on ice. The live/dead cell‐staining dye was added to each tube, mixed well, and incubated on ice in the dark for 30 min. After incubation, the cells were centrifuged at 1500 rpm for 5 min, and 5 µL of Fc block antibody and 100 µL of FACS Buffer were added to each sample tube to resuspend the cells. The mixture was then incubated on ice in the dark for 5 min. The surface antibodies were added to each tube, mixed thoroughly, and incubated on ice for 30 min. The cells were then fixed with paraformaldehyde for more than 40 min, and 100 µL of permeabilization reagent was added. The mixture was then incubated on ice for 30 min, followed by the addition of IFN‐γ antibody for intracellular staining and incubation on ice in the dark for 30 min. The cells were then centrifuged at 300 g for 5 min at room temperature, the supernatant was discarded, and the cells were resuspended in 200 µL of FACS buffer for flow cytometry analysis.

### Single‐Cell RNA‐Sequencing

Sequencing libraries were constructed using a single‐cell sequencing kit according to the manufacturer's instructions. Briefly, PBMCs were isolated, and cell viability and density were assessed by cell counting to ensure that samples with viability exceeding 80% proceeded to subsequent sequencing steps. Subsequently, the cell suspension density was adjusted to 300–600 live cells/µL and loaded onto a 10× Chromium single‐cell preparation instrument. Each single cell was lysed and reverse transcribed, followed by cDNA generation and amplification. A subsequent quality assessment was conducted to construct RNA‐Seq libraries. Finally, the RNA‐Seq libraries were sequenced using an Illumina NovaSeq 6000 (Illumina, Inc., San Diego, California, USA) sequencer, ensuring a sequencing depth of at least 1.1 million reads per cell.

### Cell Type Annotation in Single‐Cell Genomics

The Find All Markers function in Seurat was utilized to compute the differentially expressed genes within each cell cluster, which were then considered potential marker genes for identifying cell types. Subsequently, the cell types were determined based on previously published studies containing marker genes specific to each type. Cells expressing multiple marker genes for different cell types were considered doublets and excluded from further analysis. Specifically, CD3D, CD3E, and CD3G were used for T cell annotation; CD19, MS4A1, and CD79A for B cell annotation; CD14, LYZ, and CD68 for monocyte annotation; TRGV9 and TRGV2 for T cell annotation; KLRB1 for NK cell annotation; CD3D, GNLY, and KLRB1 for NKT cell annotation; and PPBP for platelet annotation.

### Definition of feature gene score

Based on literature‐reported cytotoxicity and functional exhaustion‐related genes,^[^ ^64^ ^]^ UCell V.1.1.0 was utilized to compute the cytotoxicity and functional exhaustion scores. This included six functionally impaired genes (PDCD1, CTLA‐4, TIGIT, HAVCR2, LAG3, and LAYN) and eight cytotoxicity markers (CX3CR1, PRF1, GZMA, GZMB, GZMH, GNLY, KLRG1, and NKG7).

### Isolation ETASTs from PBMC

PBMCs were cultured in a 24‐well plates for 3–4 h, and NPs (0.8 mg mL^−1^) were added to each well, followed by incubating at 37 °C for 48 h. After incubation, each sample was labeled with live/dead dye, Fc block, and membrane surface antibodies (CD3 and CD137), and ETASTs were sorted using flow cytometry in an ultra‐clean stage.

### Expanding of Isolated ETASTs or Unsorted Whole CD3^+^ T Cells

ETASTs (CD137^+^) or unsorted whole CD3^+^ T cells were collected, washed twice with PBS, and centrifuged at 350 × g for 5 min. The supernatant was discarded, and the T‐cell precipitate was resuspended. The collected T cells were cultured in a plate pre‐coated with αCD3 antibody (5 µg mL^−1^) in RPMI‐1640 complete culture medium, containing 5 µg mL^−1^ αCD28 antibody and 10 ng mL^−1^ IL‐2. Semi‐medium exchanges were performed 2–3 days.

### Isolation and Expansion of Lung Cancer Cells from Tumor Tissue of Lung Cancer Patients

The obtained tumor tissue was rinsed with DMEM/F12 (1:1) culture medium (without FBS) to remove any blood stains and placed in a culture dish. Tumor tissues were cut into small pieces (≤ 2 mm) using scissors or surgical knives, and 2–3 mL of digestive solution (containing collagenase I and II in DMEM/F12 (1:1) culture medium) were added and incubated at 37 °C for 35 min. The samples were then centrifuged, and the supernatant was discarded. The precipitate was resuspended in DMEM/F12 (1:1) culture medium and filtered through a 70 µm cell strainer to prepare a single cell suspension, followed by red blood cell lysis. The cells were then counted and cultured in a 24‐well plate with growth factors (EGF: 20 ng mL^−1^, FGF: 20 ng mL^−1^, Hydrocortisone: 50 ng mL^−1^). The cells were initially incubated in a low oxygen incubator (37 °C, 5% CO_2_, 3% O_2_) for 10 days. After that, the cells were transferred and incubated in a conventional incubator (37 °C, 5% CO_2_). Cancer cells were identified using panCK and Ki67 staining. If the isolated and expanded cells were panCK^+^ and Ki67^+^, they were considered lung cancer cells.

### Cytotoxicity of ETASTs Against Cancer Cells In Vitro

Isolated CD3^+^T cells or isolated ETASTs (CD137^+^) were mixed with the corresponding cancer cells (autologous lung cancer cells from patients or allogeneic lung cancer cells) at a ratio of 20:1. The mixture was incubated in cell culture medium for 12 or 24 h. The supernatant and non‐adherent cells (including T cells and apoptotic cells) were removed. The number of live adherent tumor cells was recorded and analyzed using a CCK8 kit. After adding the CCK8 reagents, a plate reader was used to detect the absorbance (OD value) at a wavelength of 450 nm. Cell survival rate = (As Ab)/(Ac Ab) × 100% (As: experimental absorbance well (effector cells + target) Cells; Ac: Control absorbance pore (target cell); Ab: Blank absorbance well (containing only the same culture medium]).

### Statistical Analysis

All statistical analyses were performed using GraphPad Prism software version 8.3.0 and R software version 4.1. Receiver operating characteristic (ROC) curves were plotted, and the area under the curve (AUC) was calculated to assess the predictive performance of the biomarkers for immunotherapy response. The optimal cutoff value was determined based on the ROC curve and Youden's index (Youden's index = sensitivity ^+^ specificity – 1), with the optimal cutoff value chosen as the point with the maximum Youden's index. The patients were categorized into high‐ and low‐level groups based on the optimal cutoff value. Kaplan‐Meier curves were generated using the Kaplan‐Meier method, and differences in PFS among the different groups were assessed using the log‐rank test. Data were analyzed using non‐parametric tests (Mann‐Whitney U test), t‐tests, and chi‐square tests based on data type and normality distribution. The error bars represent the standard error of the mean (SEM). Statistical p‐value < 0.05 was considered statistically significant: ^*^ p < 0.05, ^**^p < 0.01, ^***^p < 0.001. Unless otherwise stated, asterisks indicate statistical comparisons with the control group.

## Conflict of Interest

The authors declare no conflict of interest.

## Author Contributions

W.Z., J.W., Z.C. and J.Y. contributed equally to the work. M.L. and J. Z. designed the experiments, provided resources to conduct the experiments, supervised the studies, and analyzed the results of the studies. W.Z., J.W., Z.C., and J. Y. collected blood and performed T‐cell detection. W.Z., J.W., A.Z., Y.Z., and X. C. performed animal experiments. M.L., A.Z., Y.Z., X.C., and Y.L. prepared and characterized nanoparticles used in this study. W.Z., L.W., Y.X., S.J., J.C., and J.W. performed the data summary and data analysis, and W.Z., C.D., X.T., and C.L. wrote the draft of the manuscript. M.L., X.T. and J.Z. revised the manuscript.

## Supporting information



Supporting Information

## Data Availability

The data that support the findings of this study are available from the corresponding author upon reasonable request.

## References

[1] R. L. Siegel , A. N. Giaquinto , A. Jemal , CA Cancer J. Clin. 2024, 74, 12. 38230766 10.3322/caac.21820

[2] P. Sharma , J. P. Allison , Science 2015, 348, 56. 25838373 10.1126/science.aaa8172

[3] M. D. Hellmann , N. A. Rizvi , J. W. Goldman , S. N. Gettinger , H. Borghaei , J. R. Brahmer , N. E. Ready , D. E. Gerber , L. Q. Chow , R. A. Juergens , F. A. Shepherd , S. A. Laurie , W. J. Geese , S. Agrawal , T. C. Young , X. Li , S. J. Antonia , Lancet Oncol. 2017, 18, 31. 27932067 10.1016/S1470-2045(16)30624-6 PMC5476941

[4] J. R. Brahmer , S. S. Tykodi , L. Q. Chow , W. J. Hwu , S. L. Topalian , P. Hwu , C. G. Drake , L. H. Camacho , J. Kauh , K. Odunsi , H. C. Pitot , O. Hamid , S. Bhatia , R. Martins , K. Eaton , S. Chen , T. M. Salay , S. Alaparthy , J. F. Grosso , A. J. Korman , S. M. Parker , S. Agrawal , S. M. Goldberg , D. M. Pardoll , A. Gupta , J. M. Wigginton , N. Engl. J. Med. 2012, 366, 2455. 22658128 10.1056/NEJMoa1200694 PMC3563263

[5] M. Reck , J. Remon , M. D. Hellmann , J. Clin. Oncol. 2022, 40, 586. 34985920 10.1200/JCO.21.01497

[6] J. R. Brahmer , C. G. Drake , I. Wollner , J. D. Powderly , J. Picus , W. H. Sharfman , E. Stankevich , A. Pons , T. M. Salay , T. L. McMiller , M. M. Gilson , C. Wang , M. Selby , J. M. Taube , R. Anders , L. Chen , A. J. Korman , D. M. Pardoll , I. Lowy , S. L. Topalian , J. Clin. Oncol. 2010, 28, 3167. 20516446 10.1200/JCO.2009.26.7609 PMC4834717

[7] S. L. Topalian , M. Sznol , D. F. McDermott , H. M. Kluger , R. D. Carvajal , W. H. Sharfman , J. R. Brahmer , D. P. Lawrence , M. B. Atkins , J. D. Powderly , P. D. Leming , E. J. Lipson , I. Puzanov , D. C. Smith , J. M. Taube , J. M. Wigginton , G. D. Kollia , A. Gupta , D. M. Pardoll , J. A. Sosman , F. S. Hodi , J. Clin. Oncol. 2014, 32, 1020. 24590637 10.1200/JCO.2013.53.0105 PMC4811023

[8] J. M. Taube , R. A. Anders , G. D. Young , H. Xu , R. Sharma , T. L. McMiller , S. Chen , A. P. Klein , D. M. Pardoll , S. L. Topalian , L. Chen , Sci. Transl. Med. 2012, 4, 127ra37. 10.1126/scitranslmed.3003689 PMC3568523 22461641

[9] D. P. Carbone , M. Reck , L. Paz‐Ares , B. Creelan , L. Horn , M. Steins , E. Felip , M. M. van den Heuvel , T. E. Ciuleanu , F. Badin , N. Ready , T. J. N. Hiltermann , S. Nair , R. Juergens , S. Peters , E. Minenza , J. M. Wrangle , D. Rodriguez‐Abreu , H. Borghaei , G. R. Blumenschein , L. C. Villaruz , L. Havel , J. Krejci , J. Corral Jaime , H. Chang , W. J. Geese , P. Bhagavatheeswaran , A. C. Chen , M. A. Socinski , N. Engl. J. Med. 2017, 376, 2415. 28636851 10.1056/NEJMoa1613493 PMC6487310

[10] R. Cristescu , R. Mogg , M. Ayers , A. Albright , E. Murphy , J. Yearley , X. Sher , X. Q. Liu , H. Lu , M. Nebozhyn , C. Zhang , J. K. Lunceford , A. Joe , J. Cheng , A. L. Webber , N. Ibrahim , E. R. Plimack , P. A. Ott , T. Y. Seiwert , A. Ribas , T. K. McClanahan , J. E. Tomassini , A. Loboda , D. Kaufman , Science 2018, 362, aar3593. 10.1126/science.aar3593 PMC6718162 30309915

[11] S. Diem , O. Hasan Ali , C. J. Ackermann , D. Bomze , V. H. Koelzer , W. Jochum , D. E. Speiser , K. D. Mertz , L. Flatz , Cancer Immunol. Immunother. 2018, 67, 39. 28894934 10.1007/s00262-017-2061-4 PMC11028172

[12] D. T. Le , J. N. Uram , H. Wang , B. R. Bartlett , H. Kemberling , A. D. Eyring , A. D. Skora , B. S. Luber , N. S. Azad , D. Laheru , B. Biedrzycki , R. C. Donehower , A. Zaheer , G. A. Fisher , T. S. Crocenzi , J. J. Lee , S. M. Duffy , R. M. Goldberg , A. de la Chapelle , M. Koshiji , F. Bhaijee , T. Huebner , R. H. Hruban , L. D. Wood , N. Cuka , D. M. Pardoll , N. Papadopoulos , K. W. Kinzler , S. Zhou , et al., N. Engl. J. Med. 2015, 372, 2509. 26028255 10.1056/NEJMoa1500596 PMC4481136

[13] N. A. Rizvi , M. D. Hellmann , A. Snyder , P. Kvistborg , V. Makarov , J. J. Havel , W. Lee , J. Yuan , P. Wong , T. S. Ho , M. L. Miller , N. Rekhtman , A. L. Moreira , F. Ibrahim , C. Bruggeman , B. Gasmi , R. Zappasodi , Y. Maeda , C. Sander , E. B. Garon , T. Merghoub , J. D. Wolchok , T. N. Schumacher , T. A. Chan , Science 2015, 348, 124. 25765070 10.1126/science.aaa1348 PMC4993154

[14] A. Ribas , S. Hu‐Lieskovan , J. Exp. Med. 2016, 213, 2835. 27903604 10.1084/jem.20161462 PMC5154949

[15] M. Rijnders , A. A. M. van der Veldt , T. C. M. Zuiverloon , K. Grünberg , E. Thunnissen , R. de Wit , G. van Leenders , Eur. Urol. 2019, 75, 538. 30497882 10.1016/j.eururo.2018.11.002

[16] K. E. Kortekaas , S. J. Santegoets , G. Sturm , I. Ehsan , S. L. van Egmond , F. Finotello , Z. Trajanoski , M. J. P. Welters , M. I. E. van Poelgeest , S. H. van der Burg , Cancer Immunol. Res. 2020, 8, 1311. 32759363 10.1158/2326-6066.CIR-20-0270

[17] P. Savas , B. Virassamy , C. Ye , A. Salim , C. P. Mintoff , F. Caramia , R. Salgado , D. J. Byrne , Z. L. Teo , S. Dushyanthen , A. Byrne , L. Wein , S. J. Luen , C. Poliness , S. S. Nightingale , A. S. Skandarajah , D. E. Gyorki , C. M. Thornton , P. A. Beavis , S. B. Fox , P. K. Darcy , T. P. Speed , L. K. Mackay , P. J. Neeson , S. Loi , Nat. Med. 2018, 24, 986. 29942092 10.1038/s41591-018-0078-7

[18] A. M. Jaeger , L. E. Stopfer , R. Ahn , E. A. Sanders , D. A. Sandel , W. A. Freed‐Pastor , W. M. Rideout , S. Naranjo , T. Fessenden , K. B. Nguyen , P. S. Winter , R. E. Kohn , P. M. K. Westcott , J. M. Schenkel , S. L. Shanahan , A. K. Shalek , S. Spranger , F. M. White , T. Jacks , Nature 2022, 607, 149. 35705813 10.1038/s41586-022-04839-2 PMC9945857

[19] C. Puig‐Saus , B. Sennino , S. Peng , C. L. Wang , Z. Pan , B. Yuen , B. Purandare , D. An , B. B. Quach , D. Nguyen , H. Xia , S. Jilani , K. Shao , C. McHugh , J. Greer , P. Peabody , S. Nayak , J. Hoover , S. Said , K. Jacoby , O. Dalmas , S. P. Foy , A. Conroy , M. C. Yi , C. Shieh , W. Lu , K. Heeringa , Y. Ma , S. Chizari , M. J. Pilling , et al., Nature 2023, 615, 697. 36890230 10.1038/s41586-023-05787-1 PMC10441586

[20] M. M. Gubin , X. Zhang , H. Schuster , E. Caron , J. P. Ward , T. Noguchi , Y. Ivanova , J. Hundal , C. D. Arthur , W.‐J. Krebber , G. E. Mulder , M. Toebes , M. D. Vesely , S. S. K. Lam , A. J. Korman , J. P. Allison , G. J. Freeman , A. H. Sharpe , E. L. Pearce , T. N. Schumacher , R. Aebersold , H.‐G. Rammensee , C. J. M. Melief , E. R. Mardis , W. E. Gillanders , M. N. Artyomov , R. D. Schreiber , Nature 2014, 515, 577. 25428507 10.1038/nature13988 PMC4279952

[21] N. McGranahan , A. J. Furness , R. Rosenthal , S. Ramskov , R. Lyngaa , S. K. Saini , M. Jamal‐Hanjani , G. A. Wilson , N. J. Birkbak , C. T. Hiley , T. B. Watkins , S. Shafi , N. Murugaesu , R. Mitter , A. U. Akarca , J. Linares , T. Marafioti , J. Y. Henry , E. M. Van Allen , D. Miao , B. Schilling , D. Schadendorf , L. A. Garraway , V. Makarov , N. A. Rizvi , A. Snyder , M. D. Hellmann , T. Merghoub , J. D. Wolchok , S. A. Shukla , et al., Science 2016, 351, 1463. 26940869 10.1126/science.aaf1490 PMC4984254

[22] K. E. Pauken , E. J. Wherry , Trends Immunol. 2015, 36, 265. 25797516 10.1016/j.it.2015.02.008 PMC4393798

[23] D. S. Thommen , T. N. Schumacher , Cancer Cell 2018, 33, 547. 29634943 10.1016/j.ccell.2018.03.012 PMC7116508

[24] D. S. Chen , I. Mellman , Immunity 2013, 39, 1. 23890059 10.1016/j.immuni.2013.07.012

[25] H. Komuro , S. Shinohara , Y. Fukushima , A. Demachi‐Okamura , D. Muraoka , K. Masago , T. Matsui , Y. Sugita , Y. Takahashi , R. Nishida , C. Takashima , T. Ohki , Y. Shigematsu , F. Watanabe , K. Adachi , T. Fukuyama , H. Hamana , H. Kishi , D. Miura , Y. Tanaka , K. Onoue , K. Onoguchi , Y. Yamashita , R. Stratford , T. Clancy , R. Yamaguchi , H. Kuroda , K. Doi , H. Iwata , H. Matsushita , J. Immunother. Cancer 2023, 11, e007180. 37544663 10.1136/jitc-2023-007180 PMC10407349

[26] H. Kagamu , S. Kitano , O. Yamaguchi , K. Yoshimura , K. Horimoto , M. Kitazawa , K. Fukui , A. Shiono , A. Mouri , F. Nishihara , Y. Miura , K. Hashimoto , Y. Murayama , K. Kaira , K. Kobayashi , Cancer Immunol. Res. 2020, 8, 334. 31871122 10.1158/2326-6066.CIR-19-0574

[27] M. Guilliams , F. Ginhoux , C. Jakubzick , S. H. Naik , N. Onai , B. U. Schraml , E. Segura , R. Tussiwand , S. Yona , Nat. Rev. Immunol. 2014, 14, 571. 25033907 10.1038/nri3712 PMC4638219

[28] L. Ma , L. Diao , Z. Peng , Y. Jia , H. Xie , B. Li , J. Ma , M. Zhang , L. Cheng , D. Ding , X. Zhang , H. Chen , F. Mo , H. Jiang , G. Xu , F. Meng , Z. Zhong , M. Liu , Adv. Mater. 2021, 33, 2104849. 10.1002/adma.202104849 34536044

[29] L. Diao , M. Liu , Adv. Sci. (Weinh) 2023, 10, e2300121. 37254712 10.1002/advs.202300121 PMC10401146

[30] L. Diao , L. Ma , J. Cheng , Y. Pan , Z. Peng , L. Zhang , M. Xu , Y. Li , X. Zhang , H. Jiang , G. Xu , F. Meng , Z. Zhong , M. Liu , iScience 2022, 25, 105511. 36437877 10.1016/j.isci.2022.105511 PMC9682363

[31] M. E. Lutsiak , D. R. Robinson , C. Coester , G. S. Kwon , J. Samuel , Pharm. Res. 2002, 19, 1480. 12425465 10.1023/a:1020452531828

[32] S. Kumagai , Y. Togashi , T. Kamada , E. Sugiyama , H. Nishinakamura , Y. Takeuchi , K. Vitaly , K. Itahashi , Y. Maeda , S. Matsui , T. Shibahara , Y. Yamashita , T. Irie , A. Tsuge , S. Fukuoka , A. Kawazoe , H. Udagawa , K. Kirita , K. Aokage , G. Ishii , T. Kuwata , K. Nakama , M. Kawazu , T. Ueno , N. Yamazaki , K. Goto , M. Tsuboi , H. Mano , T. Doi , K. Shitara , et al., Nat. Immunol. 2020, 21, 1346. 32868929 10.1038/s41590-020-0769-3

[33] S. J. Bagley , S. Kothari , C. Aggarwal , J. M. Bauml , E. W. Alley , T. L. Evans , J. A. Kosteva , C. A. Ciunci , P. E. Gabriel , J. C. Thompson , S. Stonehouse‐Lee , V. E. Sherry , E. Gilbert , B. Eaby‐Sandy , F. Mutale , G. DiLullo , R. B. Cohen , A. Vachani , C. J. Langer , Lung Cancer 2017, 106, 1. 28285682 10.1016/j.lungcan.2017.01.013

[34] C. Putzu , D. L. Cortinovis , F. Colonese , S. Canova , C. Carru , A. Zinellu , P. Paliogiannis , Cancer Immunol. Immunother. 2018, 67, 1349. 29947960 10.1007/s00262-018-2182-4 PMC11028046

[35] A. E. Soyano , B. Dholaria , J. A. Marin‐Acevedo , N. Diehl , D. Hodge , Y. Luo , R. Manochakian , S. Chumsri , A. Adjei , K. L. Knutson , Y. Lou , J. Immunother. Cancer 2018, 6, 129. 30470260 10.1186/s40425-018-0447-2 PMC6251165

[36] J. M. Riedl , D. A. Barth , W. M. Brueckl , G. Zeitler , V. Foris , S. Mollnar , M. Stotz , C. H. Rossmann , A. Terbuch , M. Balic , T. Niedrist , T. Bertsch , H. Stoeger , M. Pichler , H. Olschewski , G. Absenger , J. H. Ficker , A. Gerger , F. Posch , Cancers (Basel) 2020, 12, 2319. 32824580 10.3390/cancers12082319 PMC7464328

[37] A. Costantini , C. Julie , C. Dumenil , Z. Hélias‐Rodzewicz , J. Tisserand , J. Dumoulin , V. Giraud , S. Labrune , T. Chinet , J.‐F. Emile , E. G. Leprieur , Oncoimmunology 2018, 7, e1452581. 30221046 10.1080/2162402X.2018.1452581 PMC6136870

[38] Y. K. Cheng , D. W. Chen , P. Chen , X. He , P. S. Li , Z. S. Lin , S. X. Chen , S. B. Ye , P. Lan , Front. Immunol. 2022, 13, 809971. 35185898 10.3389/fimmu.2022.809971 PMC8850282

[39] K. E. Pauken , O. Shahid , K. A. Lagattuta , K. M. Mahuron , J. M. Luber , M. M. Lowe , L. Huang , C. Delaney , J. M. Long , M. E. Fung , K. Newcomer , K. K. Tsai , M. Chow , S. Guinn , J. R. Kuchroo , K. P. Burke , J. M. Schenkel , M. D. Rosenblum , A. I. Daud , A. H. Sharpe , M. Singer , J. Exp. Med. 2021, 218, 20200920. 10.1084/jem.20200920 PMC7933992 33651880

[40] G. Oliveira , K. Stromhaug , S. Klaeger , T. Kula , D. T. Frederick , P. M. Le , J. Forman , T. Huang , S. Li , W. Zhang , Q. Xu , N. Cieri , K. R. Clauser , S. A. Shukla , D. Neuberg , S. Justesen , G. MacBeath , S. A. Carr , E. F. Fritsch , N. Hacohen , M. Sade‐Feldman , K. J. Livak , G. M. Boland , P. A. Ott , D. B. Keskin , C. J. Wu , Nature 2021, 596, 119. 34290406 10.1038/s41586-021-03704-y PMC9187974

[41] A. Ribas , J. D. Wolchok , Science 2018, 359, 1350. 29567705 10.1126/science.aar4060 PMC7391259

[42] P. I. Gonzalez‐Ericsson , E. S. Stovgaard , L. F. Sua , E. Reisenbichler , Z. Kos , J. M. Carter , S. Michiels , J. Le Quesne , T. O. Nielsen , A.‐V. Lænkholm , S. B. Fox , J. Adam , J. M. Bartlett , D. L. Rimm , C. Quinn , D. Peeters , M. V. Dieci , A. Vincent‐Salomon , I. Cree , A. I. Hida , J. M. Balko , H. R. Haynes , I. Frahm , G. Acosta‐Haab , M. Balancin , E. Bellolio , W. Yang , P. Kirtani , T. Sugie , A. Ehinger , et al., J. Pathol. 2020, 250, 667. 32129476 10.1002/path.5406

[43] K. H. Kim , C. G. Kim , E. C. Shin , Immune Netw. 2020, 20, e8. 32158596 10.4110/in.2020.20.e8 PMC7049582

[44] L. Hutchinson , Nat. Rev. Clin. Oncol. 2016, 13, 203. 26977781 10.1038/nrclinonc.2016.38

[45] A. O. Kamphorst , R. N. Pillai , S. Yang , T. H. Nasti , R. S. Akondy , A. Wieland , G. L. Sica , K. Yu , L. Koenig , N. T. Patel , M. Behera , H. Wu , M. McCausland , Z. Chen , C. Zhang , F. R. Khuri , T. K. Owonikoko , R. Ahmed , S. S. Ramalingam , Proc. Natl. Acad. Sci. U S A 2017, 114, 4993. 28446615 10.1073/pnas.1705327114 PMC5441721

[46] I. G. Zizzari , A. Di Filippo , A. Botticelli , L. Strigari , A. Pernazza , E. Rullo , M. G. Pignataro , A. Ugolini , F. Scirocchi , F. R. Di Pietro , E. Rossi , A. Gelibter , G. Schinzari , G. D'Amati , A. Rughetti , P. Marchetti , M. Nuti , C. Napoletano , Clin. Cancer Res. 2022, 28, 1027. 34980602 10.1158/1078-0432.CCR-21-2918 PMC9377756

[47] A. Gros , M. R. Parkhurst , E. Tran , A. Pasetto , P. F. Robbins , S. Ilyas , T. D. Prickett , J. J. Gartner , J. S. Crystal , I. M. Roberts , K. Trebska‐McGowan , J. R. Wunderlich , J. C. Yang , S. A. Rosenberg , Nat. Med. 2016, 22, 433. 26901407 10.1038/nm.4051 PMC7446107

[48] V. Shankaran , H. Ikeda , A. T. Bruce , J. M. White , P. E. Swanson , L. J. Old , R. D. Schreiber , Nature 2001, 410, 1107.11323675

[49] Y. Simoni , E. Becht , M. Fehlings , C. Y. Loh , S. L. Koo , K. W. W. Teng , J. P. S. Yeong , R. Nahar , T. Zhang , H. Kared , K. Duan , N. Ang , M. Poidinger , Y. Y. Lee , A. Larbi , A. J. Khng , E. Tan , C. Fu , R. Mathew , M. Teo , W. T. Lim , C. K. Toh , B. H. Ong , T. Koh , A. M. Hillmer , A. Takano , T. K. H. Lim , E. H. Tan , W. Zhai , D. S. W. Tan , et al., Nature 2018, 557, 575. 29769722 10.1038/s41586-018-0130-2

[50] N. A. Giraldo , E. Becht , Y. Vano , F. Petitprez , L. Lacroix , P. Validire , R. Sanchez‐Salas , A. Ingels , S. Oudard , A. Moatti , B. Buttard , S. Bourass , C. Germain , X. Cathelineau , W. H. Fridman , C. Sautès‐Fridman , Clin. Cancer Res. 2017, 23, 4416. 28213366 10.1158/1078-0432.CCR-16-2848

[51] X. He , C. Xu , Cell Res. 2020, 30, 660. 32467592 10.1038/s41422-020-0343-4 PMC7395714

[52] A. Ribas , J. D. Wolchok , Science 2017, 359, 1350. 10.1126/science.aar4060 PMC7391259 29567705

[53] K. E. Pauken , E. J. Wherry , Trends Immunol. 2015, 36, 265. 25797516 10.1016/j.it.2015.02.008 PMC4393798

[54] A. Chow , K. Perica , C. A. Klebanoff , J. D. Wolchok , Nat. Rev. Clin. Oncol. 2022, 19, 775. 36216928 10.1038/s41571-022-00689-z PMC10984554

[55] S. Lu , J. E. Stein , D. L. Rimm , D. W. Wang , J. M. Bell , D. B. Johnson , J. A. Sosman , K. A. Schalper , R. A. Anders , H. Wang , C. Hoyt , D. M. Pardoll , L. Danilova , J. M. Taube , JAMA Oncol. 2019, 5, 1195. 31318407 10.1001/jamaoncol.2019.1549 PMC6646995

[56] L. Q. M. Chow , R. Haddad , S. Gupta , A. Mahipal , R. Mehra , M. Tahara , R. Berger , J. P. Eder , B. Burtness , S. H. Lee , B. Keam , H. Kang , K. Muro , J. Weiss , R. Geva , C. C. Lin , H. C. Chung , A. Meister , M. Dolled‐Filhart , K. Pathiraja , J. D. Cheng , T. Y. Seiwert , J. Clin. Oncol. 2016, 34, 3838. 27646946 10.1200/JCO.2016.68.1478 PMC6804896

[57] A. B. El‐Khoueiry , B. Sangro , T. Yau , T. S. Crocenzi , M. Kudo , C. Hsu , T. Y. Kim , S. P. Choo , J. Trojan , T. H. R. Welling , T. Meyer , Y. K. Kang , W. Yeo , A. Chopra , J. Anderson , C. Dela Cruz , L. Lang , J. Neely , H. Tang , H. B. Dastani , I. Melero , Lancet 2017, 389, 2492. 28434648 10.1016/S0140-6736(17)31046-2 PMC7539326

[58] T. D. Wu , S. Madireddi , P. E. de Almeida , R. Banchereau , Y.‐J. J. Chen , A. S. Chitre , E. Y. Chiang , H. Iftikhar , W. E. O'Gorman , A. Au‐Yeung , C. Takahashi , L. D. Goldstein , C. Poon , S. Keerthivasan , D. E. de Almeida Nagata , X. Du , H.‐M. Lee , K. L. Banta , S. Mariathasan , M. D. Thakur , M. A. Huseni , M. Ballinger , I. Estay , P. Caplazi , Z. Modrusan , L. Delamarre , I. Mellman , R. Bourgon , J. L. Grogan , Nature 2020, 579, 274. 32103181 10.1038/s41586-020-2056-8

[59] K. Van Raemdonck , P. E. Van den Steen , S. Liekens , J. Van Damme , S. Struyf , Cytokine Growth Factor Rev. 2015, 26, 311. 25498524 10.1016/j.cytogfr.2014.11.009

[60] G. Elia , P. Fallahi , Clin. Ter. 2017, 168, e37. 28240761 10.7417/CT.2017.1980

[61] L. Liu , J. Chen , H. Zhang , J. Ye , C. Moore , C. Lu , Y. Fang , Y. X. Fu , B. Li , Nat. Cancer 2022, 3, 437. 35393580 10.1038/s43018-022-00352-7 PMC9050907

[62] R. Shen , M. A. Postow , M. Adamow , A. Arora , M. Hannum , C. Maher , P. Wong , M. A. Curran , T. J. Hollmann , L. Jia , H. Al‐Ahmadie , N. Keegan , S. A. Funt , G. Iyer , J. E. Rosenberg , D. F. Bajorin , P. B. Chapman , A. N. Shoushtari , A. S. Betof , P. Momtaz , T. Merghoub , J. D. Wolchok , K. S. Panageas , M. K. Callahan , Sci. Transl. Med. 2021, 13, eabf5107. 34433638 10.1126/scitranslmed.abf5107 PMC9254663

[63] J. Larkin , V. Chiarion‐Sileni , R. Gonzalez , J. J. Grob , C. L. Cowey , C. D. Lao , D. Schadendorf , R. Dummer , M. Smylie , P. Rutkowski , P. F. Ferrucci , A. Hill , J. Wagstaff , M. S. Carlino , J. B. Haanen , M. Maio , I. Marquez‐Rodas , G. A. McArthur , P. A. Ascierto , G. V. Long , M. K. Callahan , M. A. Postow , K. Grossmann , M. Sznol , B. Dreno , L. Bastholt , A. Yang , L. M. Rollin , C. Horak , F. S. Hodi , et al., N. Engl. J. Med. 2015, 373, 23. 26027431 10.1056/NEJMoa1504030 PMC5698905

[64] A. M. van der Leun , D. S. Thommen , T. N. Schumacher , Nat. Rev. Cancer 2015, 20, 218. 10.1038/s41568-019-0235-4 PMC7115982 32024970

