# Supplementary material for: Efficient Predictor for Immunotherapy Efficacy: Detecting Pan‐Clones Effector Tumor Antigen‐Specific T Cells in Blood by Nanoparticles Loading Whole Tumor Antigens
Source: Adv Sci (Weinh). 2024 Nov 5;12(2):2409913. doi: 10.1002/advs.202409913 (PMC11727128; doi:10.1002/advs.202409913)
Supplement: Supplementary file 1 — Supporting Information [file ADVS-12-2409913-s001.docx]

**Supplementary materials**

**Efficient predictor for immunotherapy efficacy: detecting pan-clones effector tumor antigen specific T cells in blood by nanoparticles loading whole tumor antigens**

Weibiao Zeng^1,2,3,4,5#^, Jin Wang^2,5#^, Zhike Chen^1,3,5#^, Jian Yang^1,3,5#^, Ao Zhu^2,5^, Yan Zheng^2,5^, Xianlan Chen^2,5^, Yuhan Liu^2,5^, Leilei Wu^6^, Yufeng Xie^1,3,5^, Sheng Ju^1,3,5^, Jun Chen^1,3,5^, Cheng Ding^1,3,5^, Chang Li^1,3,5^, Xin Tong^1,3,5^*, Mi Liu^2,5,7,8,9^*, Jun Zhao^1,3,5^*

^1^ Institute of Thoracic Surgery, The First Affiliated Hospital of Soochow University, Soochow University, Suzhou, Jiangsu, 215123

^2^ Department of Pharmaceutics, College of Pharmaceutical Science, Soochow University, Suzhou, Jiangsu, 215123

^3^ Department of Thoracic Surgery, The First Affiliated Hospital of Soochow University, Soochow University, Suzhou, Jiangsu, 215123

^4^ Department of Thoracic Surgery, Shanghai General Hospital, Shanghai Jiaotong University School of Medicine, Shanghai, 200080, People’s Republic of China

^5^ Institute of Minimally Invasive Thoracic Cancer Therapy and Translational Research, Soochow University, Suzhou, Jiangsu, 215123, People's Republic of China

^6^ Department of Radiotherapy, Shanghai Pulmonary Hospital of Tongji University, Shanghai, China, 200000

^7^ Suzhou Ersheng Biopharmaceutical Co., Ltd., Suzhou, 215123, People's Republic of China

^8^ Jiangsu Province Engineering Research Center of Precision Diagnostics and Therapeutics Development, Soochow University, Suzhou 215123, China

^9^ Wuxi Boston Biopharmaceutical Co., Ltd., Wuxi, 214125, People's Republic of China

# Contributed equally to the work

* Corresponding Authors: Mi Liu, College of Pharmaceutical Science, Soochow University, Suzhou, Jiangsu, 215123, contact Email: [mi.liu@suda.edu.cn](mailto:mi.liu@suda.edu.cn)

Xin Tong, Institute of Thoracic Surgery, The First Affiliated Hospital of Soochow University, Soochow University, Suzhou, Jiangsu, 215123, contact Email: [tongxin900225@163.com](mailto:tongxin900225@163.com)

Jun Zhao, Institute of Thoracic Surgery, The First Affiliated Hospital of Soochow University, Soochow University, Suzhou, Jiangsu, 215123, contact Email: zhaojia0327@126.com

**Supplementary table S1**. Detailed information of nanoparticles.

| **Nanoparticles** | **Antigen Source** | **Adjuvant** | **Solution for dissolving antigens** | **Size (nm)** | **Zeta potential (mV)** | **PDI** | **Loading capacity (μg/1mg PLGA)** |
| --- | --- | --- | --- | --- | --- | --- | --- |
| Blank NP | No | No | Water | 198 ± 11 | -18.2 ± 1.3 | 0.154 ± 0.023 | 0 |
| LLCC WNP | LLC cell lysates | No | Water | 205 ± 21 | -19.3 ± 1.7 | 0.152 ± 0.041 | 71 |
| LLCC UNP | LLC cell lysates | No | 8M Urea | 211 ± 20 | -20.5 ± 1.2 | 0.157 ± 0.025 | 74 |
| LLCC WNP | LLC cell lysates | Poly IC | Water | 207 ± 22 | -19.2 ± 1.2 | 0.162 ± 0.042 | 72 |
| LLCC UNP | LLC cell lysates | Poly IC | 8M Urea | 214 ± 18 | -18.8 ± 1.8 | 0.159 ± 0.031 | 76 |
| LLCT WNP | LLC Tumor Tissue Lysate | No | Water | 209 ± 14 | -20.2 ± 1.4 | 0.162 ± 0.047 | 69 |
| LLCT UNP | LLC Tumor Tissue Lysate | No | 8M Urea | 213 ± 12 | -20.3 ± 1.2 | 0.160 ± 0.041 | 72 |
| LLCT WNP | LLC Tumor Tissue Lysate | Poly IC | Water | 204 ± 18 | -19.5 ± 1.5 | 0.159 ± 0.046 | 71 |
| LLCT UNP | LLC Tumor Tissue Lysate | Poly IC | 8M Urea | 201 ± 14 | -18.7 ± 1.9 | 0.157 ± 0.041 | 73 |
| MUC1 NP | MUC1 | No | Water | 202 ± 16 | -20.1 ± 1.2 | 0.156 ± 0.032 | 71 |
| MAP NP | MUC1, CEA, MAGE-A3 | No | Water | 204 ± 17 | -19.3 ± 1.4 | 0.155 ± 0.045 | 72 |
| A549 WNP | A549 cell lysate | No | Water | 210 ± 28 | -20.5 ± 1.5 | 0.153 ± 0.032 | 69 |
| A549 UNP | A549 cell lysate | No | 8M Urea | 211 ± 25 | -20.2 ± 1.2 | 0.157 ± 0.035 | 72 |
| mTC WNP | A549, H1299, H1650, PC9, H226, H520, SK-MES-1 cell lysate | No | Water | 208 ± 26 | -20.5 ± 1.1 | 0.162 ± 0.041 | 73 |
| mTC UNP | A549, H1299, H1650, PC9, H226, H520, SK-MES-1cell lysate | No | 8M Urea | 212 ± 17 | -19.3 ± 1.4 | 0.164 ± 0.046 | 68 |
| sTT WNP | Tumor tissue lysates from a single lung cancer patient | No | Water | 206 ± 14 | -19.9 ± 1.3 | 0.165 ± 0.032 | 73 |
| sTT UNP | Tumor tissue lysates from a single lung cancer patient | No | 8M Urea | 211 ± 18 | -19.3 ± 1.1 | 0.167 ± 0.046 | 71 |
| sTT WNP | Tumor tissue lysates from a single lung cancer patient | Poly IC | Water | 216 ± 15 | -19.6 ± 1.6 | 0.156 ± 0.031 | 69 |
| sTT UNP | Tumor tissue lysates from a single lung cancer patient | Poly IC | 8M Urea | 207 ± 17 | -20.1 ± 1.2 | 0.159 ± 0.041 | 74 |
| mTT WNP | Tumor tissue lysates from multiple lung cancer patients | No | Water | 203 ± 21 | -20.2 ± 1.1 | 0.158 ± 0.035 | 72 |
| mTT UNP | Tumor tissue lysates from multiple lung cancer patients | No | 8M Urea | 213 ± 22 | -20.4 ± 1.3 | 0.158 ± 0.042 | 67 |
| mTT WNP | Tumor tissue lysates from multiple lung cancer patients | Poly IC | Water | 206 ± 18 | -20.1 ± 1.9 | 0.157 ± 0.037 | 74 |
| mTT UNP | Tumor tissue lysates from multiple lung cancer patients | Poly IC | 8M Urea | 216 ± 21 | -19.4 ± 1.4 | 0.161 ± 0.046 | 72 |

**Supplementary table S2**. Basic clinical information of NSCLC patients.

| **Characteristics** | **Clusters** | **Number of patients** | **Percentage %** |
| --- | --- | --- | --- |
| Age-years | ≤65 | 15 | 37.50% |
|  | ＞65 | 25 | 62.50% |
| Sex | Female | 4 | 10.00% |
|  | Male | 36 | 90.00% |
| ECOG PS | 0 | 20 | 50.00% |
|  | 1 | 20 | 50.00% |
| Smoking history | Heavy | 27 | 67.50% |
|  | Never/light | 13 | 32.50% |
| Histology | Adenocarcinoma | 29 | 72.50% |
|  | Squamous cell carcinoma | 11 | 27.50% |
| Disease staging | Ⅲ | 24 | 60.00% |
|  | Ⅳ | 16 | 40.00% |
| Metastasis | Yes | 7 | 17.50% |
|  | No | 33 | 82.50% |
| PD-1 blockade | Pembrolizumab | 7 | 17.50% |
|  | Tislelizumab | 13 | 32.50% |
|  | Sintilimab | 20 | 50.00% |
| Clinical efficacy | CR/PR | 14 | 35.00% |
|  | SD | 21 | 52.50% |
|  | PD | 5 | 12.50% |
| PD-L1 TPS | ＜1% | 18 | 45.00% |
|  | 1-49% | 14 | 35.00% |
|  | ≥50% | 8 | 20.00% |

**Supplementary table S3**. Detailed clinical information of NSCLC patients.

| **Patients** | **Clinical response** | **Age** | **Histology** | **Sex** | **Disease stage** | **ECOG-PS** | **Smoking history** | **NLR** | **Platelet count (10^ 9 /L)** | **LDH (U/L)** | **CRP (mg/L)** | **BMI** | **PD-L1 TPS** |
| --- | --- | --- | --- | --- | --- | --- | --- | --- | --- | --- | --- | --- | --- |
| 1 | PR | 70 | Squamous cell carcinoma | male | Ⅲ | 1 | No-smoking | 1.895833 | 156 | 139.3 | 1.99 | 18.4 | 0 |
| 2 | SD | 65 | Adenocarcinoma | male | Ⅲ | 0 | Smoking | 6.729167 | 177 | 178.4 | 4.19 | 25 | 0 |
| 3 | SD | 74 | Squamous cell carcinoma | male | Ⅲ | 1 | No-smoking | 4.010753 | 203 | 216 | 2.28 | 24 | 0.01 |
| 4 | PD | 58 | Adenocarcinoma | female | IV | 1 | No-smoking | 3.38 | 170 | 170.5 | 11.36 | 22.43 | 0.01 |
| 5 | PD | 72 | Adenocarcinoma | male | Ⅲ | 0 | Smoking | 0.69145 | 142 | 127.8 | 1.12 | 21.47 | 0.01 |
| 6 | PR | 74 | Adenocarcinoma | male | Ⅲ | 0 | Smoking | 0.168306 | 298 | 421.9 | 15.36 | 22.1 | 0.01 |
| 7 | SD | 65 | Adenocarcinoma | male | IV | 0 | Smoking | 2.528169 | 133 | 237.7 | 6.78 | 26 | 0.01 |
| 8 | PR | 55 | Adenocarcinoma | male | Ⅲ | 1 | Smoking | 1.522167 | 112 | 226.4 | 5.48 | 19.4 | 0.01 |
| 9 | SD | 68 | Adenocarcinoma | male | Ⅲ | 0 | Smoking | 1.767241 | 191 | 239 | 2.88 | 21.48 | 0.02 |
| 10 | PR | 55 | Adenocarcinoma | male | Ⅲ | 1 | Smoking | 2.327434 | 156 | 137 | 6.39 | 25 | 0.02 |
| 11 | SD | 58 | Squamous cell carcinoma | male | Ⅲ | 1 | Smoking | 2.133758 | 164 | 182.2 | 14.55 | 20.19 | 0.02 |
| 12 | PD | 62 | Adenocarcinoma | male | IV | 0 | Smoking | 1.445255 | 106 | 266.8 | 11.38 | 22.4 | 0.03 |
| 13 | SD | 80 | Adenocarcinoma | female | Ⅲ | 1 | Smoking | 2.2 | 148 | 236 | 12.75 | 21.4 | 0.03 |
| 14 | SD | 57 | Adenocarcinoma | female | IV | 1 | Smoking | 1.619565 | 163 | 182.3 | 4.02 | 23.1 | 0.03 |
| 15 | SD | 65 | Adenocarcinoma | male | Ⅲ | 0 | No-smoking | 1.493976 | 137 | 234.7 | 14.68 | 24.22 | 0.04 |
| 16 | SD | 72 | Squamous cell carcinoma | male | IV | 0 | Smoking | 2.243573 | 216 | 178.3 | 6.24 | 18.2 | 0.04 |
| 17 | SD | 72 | Adenocarcinoma | male | IV | 0 | No-smoking | 4.581197 | 180 | 169.5 | 15.36 | 20.28 | 0.05 |
| 18 | PR | 74 | Squamous cell carcinoma | male | Ⅲ | 1 | No-smoking | 0.22878 | 230 | 216.8 | 8.54 | 19.45 | 0.05 |
| 19 | PR | 80 | Squamous cell carcinoma | male | Ⅲ | 1 | Smoking | 1.904762 | 223 | 174.8 | 6.45 | 17.6 | 0.1 |
| 20 | SD | 80 | Squamous cell carcinoma | male | Ⅲ | 1 | Smoking | 3.366071 | 138 | 217 | 4.41 | 22.8 | 0.1 |
| 21 | SD | 71 | Adenocarcinoma | male | Ⅲ | 0 | Smoking | 2.132231 | 167 | 134 | 0.56 | 19.9 | 0.1 |
| 22 | SD | 71 | Adenocarcinoma | male | Ⅲ | 1 | Smoking | 2.293233 | 235 | 198 | 2.77 | 13.5 | 0.1 |
| 23 | PD | 69 | Squamous cell carcinoma | male | IV | 1 | No-smoking | 1.306818 | 266 | 164.3 | 7.02 | 26 | 0.11 |
| 24 | SD | 63 | Adenocarcinoma | male | IV | 0 | Smoking | 3.477778 | 219 | 181 | 9.26 | 16.74 | 0.21 |
| 25 | PR | 81 | Squamous cell carcinoma | male | Ⅲ | 1 | Smoking | 2.02 | 305 | 136 | 8.08 | 23 | 0.25 |
| 26 | PR | 73 | Squamous cell carcinoma | male | Ⅲ | 1 | Smoking | 0.111842 | 105 | 145 | 3.48 | 21.2 | 0.28 |
| 27 | PR | 87 | Adenocarcinoma | male | Ⅲ | 0 | No-smoking | 0.75 | 206 | 200 | 0.96 | 17.4 | 0.35 |
| 28 | PR | 73 | Adenocarcinoma | female | IV | 1 | Smoking | 1.576271 | 182 | 171.5 | 0.95 | 18.5 | 0.32 |
| 29 | PR | 71 | Adenocarcinoma | male | IV | 1 | Smoking | 5.882353 | 217 | 146 | 15.36 | 21.48 | 0.4 |
| 30 | SD | 73 | Adenocarcinoma | male | Ⅲ | 0 | No-smoking | 1.366667 | 94 | 187.8 | 3.3 | 23 | 0.45 |
| 31 | SD | 65 | Adenocarcinoma | male | IV | 0 | No-smoking | 2.835366 | 279 | 239.2 | 4.18 | 222 | 0.35 |
| 32 | PD | 68 | Adenocarcinoma | male | IV | 1 | No-smoking | 2.835366 | 279 | 239.2 | 4.18 | 222 | 0.8 |
| 33 | SD | 72 | Adenocarcinoma | male | Ⅲ | 0 | Smoking | 3 | 1.58 | 143.8 | 6.68 | 21.25 | 0.6 |
| 34 | PR | 67 | Adenocarcinoma | male | Ⅲ | 0 | Smoking | 3.45 | 248 | 217.4 | 5.44 | 23.2 | 0.7 |
| 35 | SD | 65 | Adenocarcinoma | male | Ⅲ | 0 | Smoking | 1.898551 | 216 | 144.7 | 10.13 | 23.18 | 0.75 |
| 36 | SD | 67 | Squamous cell carcinoma | male | IV | 1 | Smoking | 2.5875 | 113 | 153 | 15.36 | 26.57 | 0.85 |
| 37 | SD | 77 | Adenocarcinoma | male | Ⅲ | 0 | No-smoking | 2.964029 | 183 | 249.3 | 13.26 | 21.96 | 0.85 |
| 38 | PR | 60 | Adenocarcinoma | male | IV | 0 | No-smoking | 1.18018 | 269 | 171.1 | 6.38 | 22.49 | 0.95 |
| 39 | SD | 62 | Adenocarcinoma | male | IV | 0 | Smoking | 3.76087 | 298 | 195.1 | 2.17 | 22 | 0.9 |
| 40 | PR | 60 | Adenocarcinoma | male | IV | 1 | Smoking | 1.659574 | 112 | 219.5 | 14.47 | 21.3 | 0.9 |

**
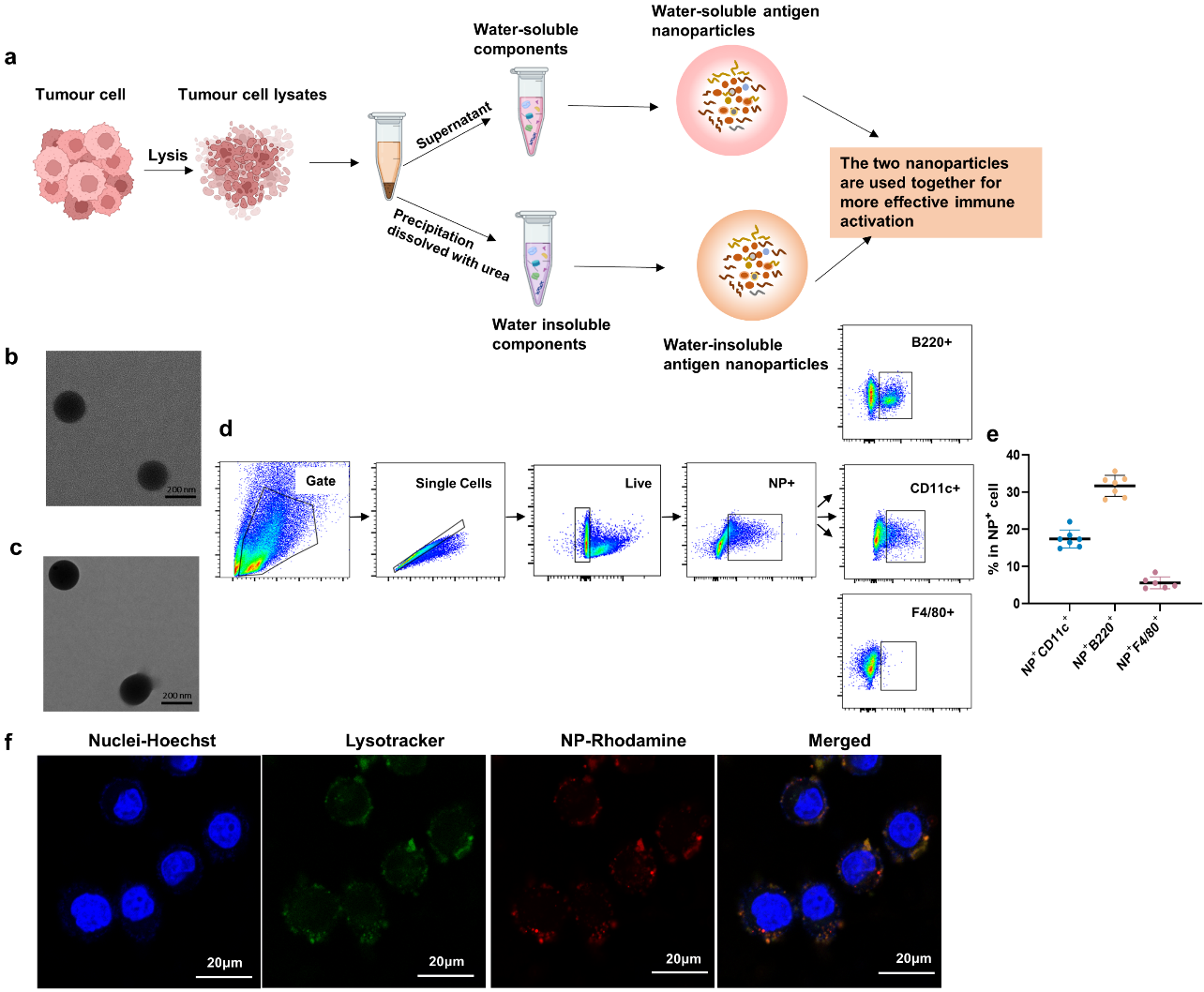
**

**Supplementary Fig. S1 Preparation and *in vitro* characterization of nanoparticles (NPs) loaded with whole tumor cell antigens.** **a**, Brief schematic diagram of the preparation of nanoparticles loaded with whole-cell tumor antigens. **b**, Electron micrograph of water-soluble antigen nanoparticles. **c**, Electron micrograph of water-insoluble antigen nanoparticles. **d**, Flow cytometry plot showing the gating strategy of nanoparticle uptake by splenocytes. **e**, Proportion of nanoparticle uptake by B-cells (B220), DC-cells (CD11c), and monocyte-macrophages (F4/80). **f**, Nanoparticle uptake by DC2.4 cells after 6 h incubation with the nanoparticles. Scale bar: 20 μm. Blue, Hoechst labeled nuclei; Green, Lysotracker Green labeled endosome-lysosomes; Red, Nanoparticles loaded with rhodamine B.


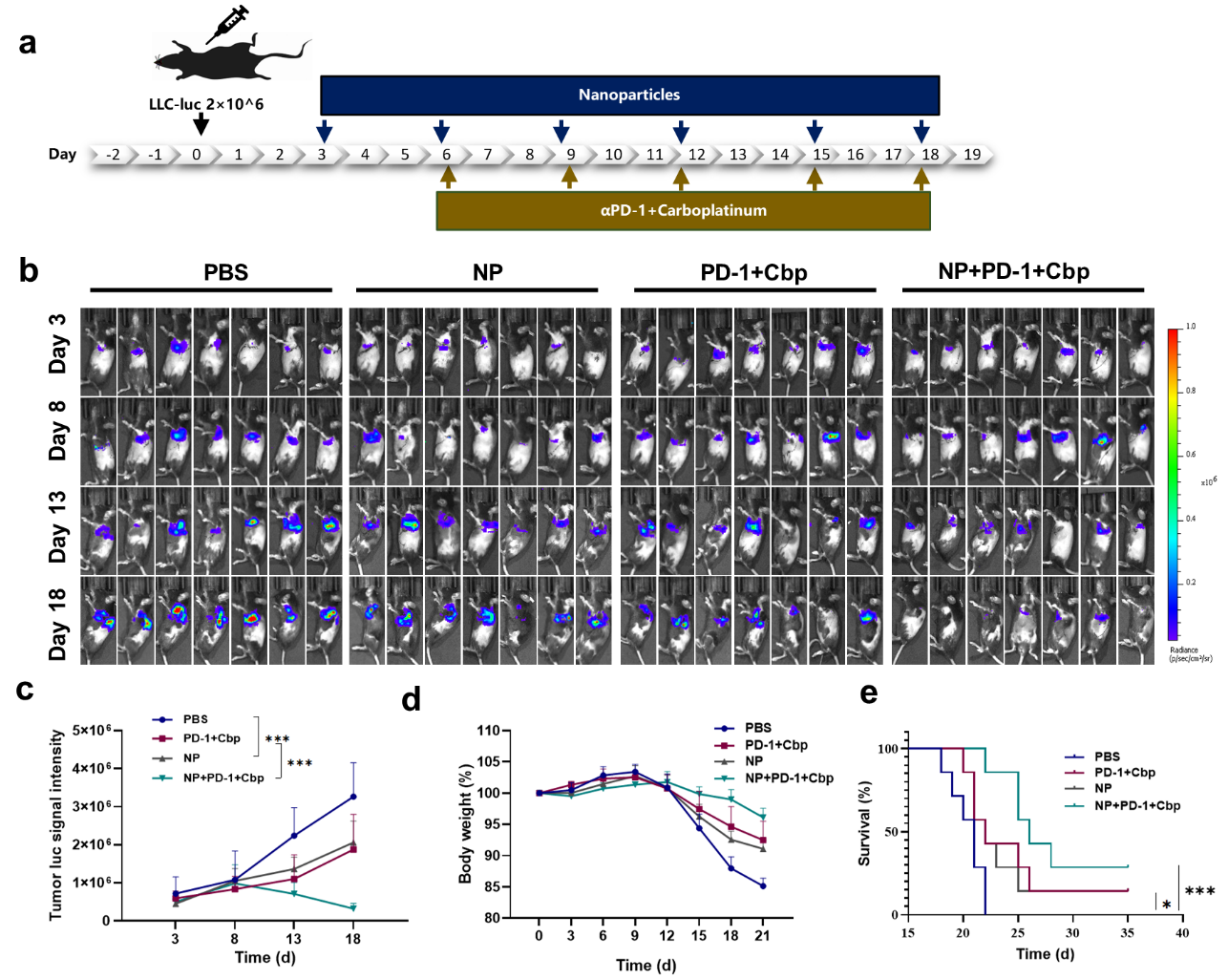


**Supplementary Fig. S2 Therapeutic efficacy of antigen NPs in an in situ LLC lung cancer mouse model. a**, Flowchart of in situ tumor inoculation and drug administration. **b**, Bioluminescence signals of in situ lung cancer observed by near-infrared in vivo imaging during the treatment period. **c**, Tumor size monitoring of in situ lung cancer during the treatment period, which was calculated based on statistical relative bioluminescence intensities. **d,** Body weight changes in each group of mice during the treatment period. **e,** Survival of mice with in situ lung cancer (n=7). Data are presented as Mean ± SEM. P-values <0.05 were considered significant: ^*^P<0.05, ^**^P<0.01, ^***^P<0.001.


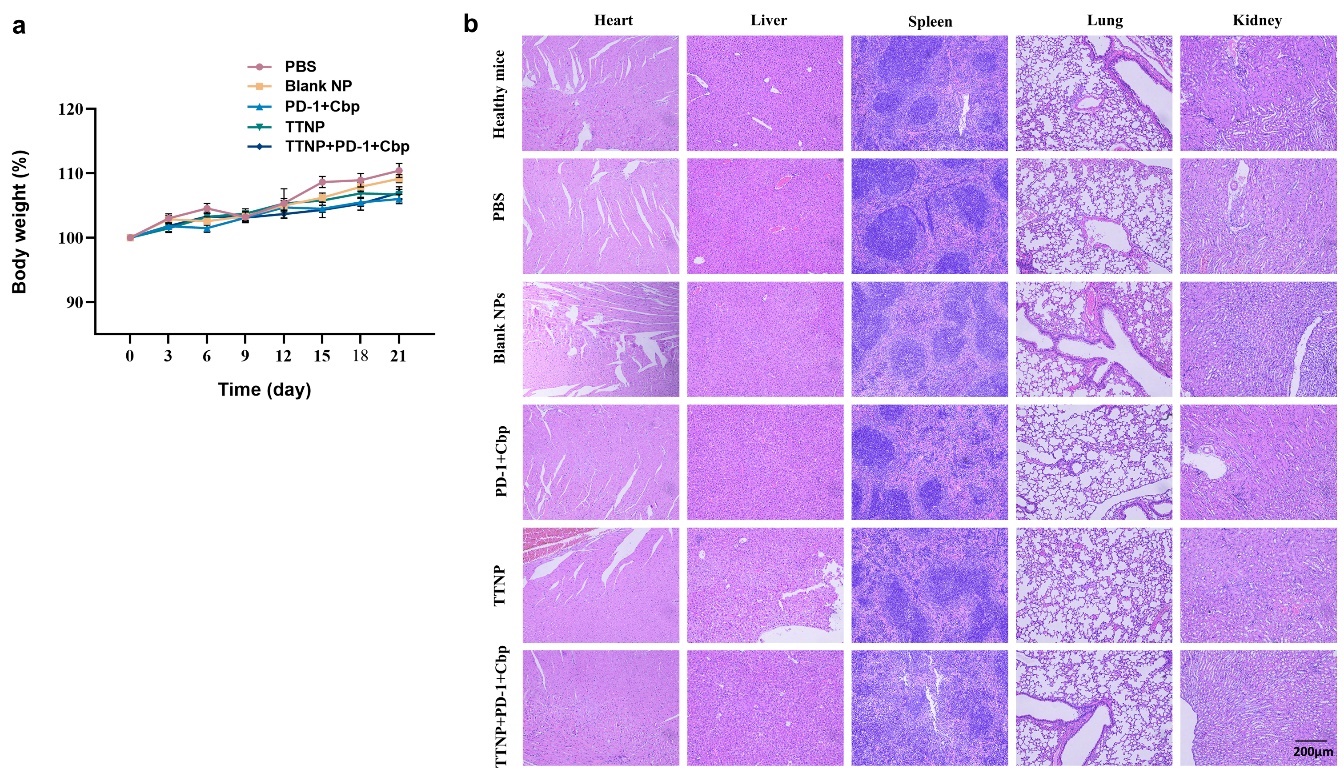


**Supplementary Fig. S3** **Biosafety evaluation of whole-antigens loaded NPs**. **a**, Weight change curve of mice during treatment. **b**, H&E staining of major organs of mice at the end of treatment. Scale bar: 200 μm.


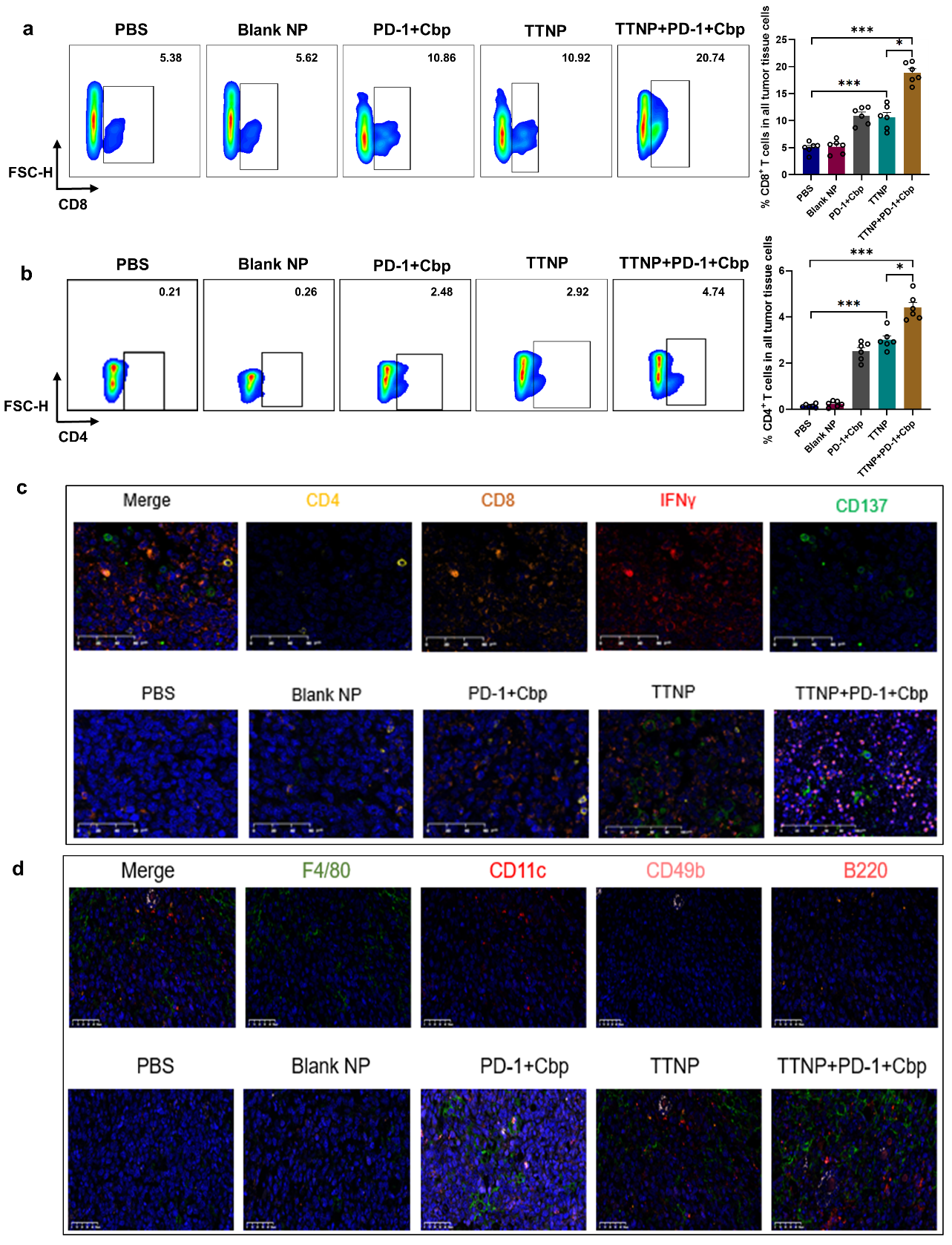


**Supplementary Fig. S4 Whole-antigens loaded NPs induce increased ETAST in the tumor microenvironment and positively correlated with therapeutic efficacy. a**，Representative flow cytometry results (left) and summary (right) of CD8^+^ T cells in tumor tissue。**b**，Representative flow cytometry results (left) and summary (right) of CD4^+^ T cells in tumor tissue. **c**, Immunofluorescence staining analysis of T cells and cytotoxic T cells in treated tumor tissues. **d**, Immunofluorescence staining analysis of CD11c (DCs), B220 (B cells), F4/80 (macrophages), and CD49b (NK cells) in treated tumor tissues. Data are presented as Mean ± SEM. P-values <0.05 were considered significant: ^*^P<0.05, ^**^P<0.01, ^***^P<0.001


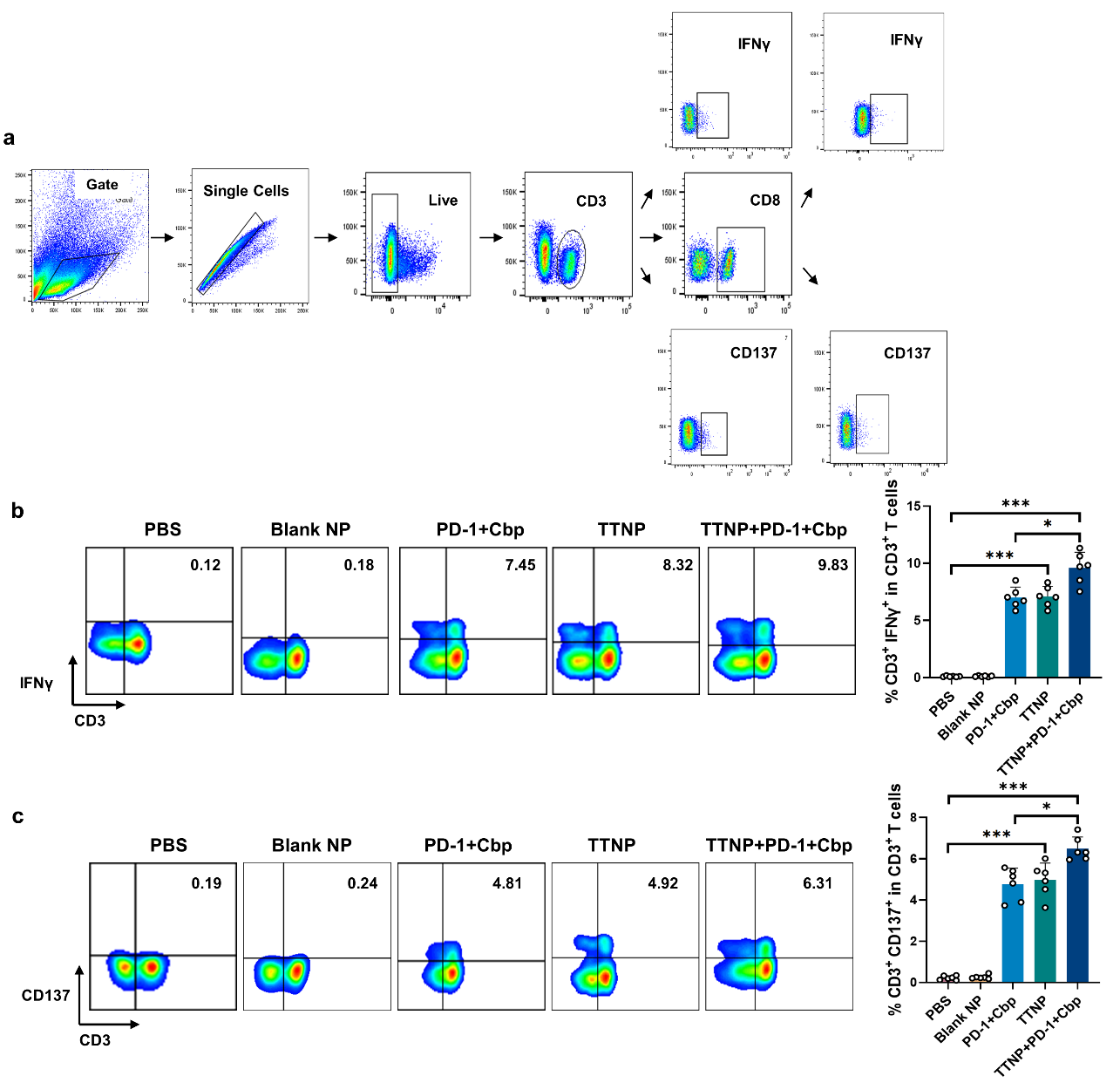


**Supplementary Fig. S5 Antigen NPs induce an increase of ETASTs in peripheral blood and the amount of ETASTs is positively correlated with treatment efficacy. a**, Flow cytometry plot showing the gating strategy of ETASTs induced by antigen NPs. **b**, Representative flow cytometry results of IFN-γ^+^ T cells (ETASTs, left) and summary (right). **c**, Representative flow cytometry results of CD137^+^ T cells (ETASTs, left) and summary (right). Data are presented as Mean ± SEM. P-values <0.05 were considered significant: ^*^P<0.05, ^**^P<0.01, ^***^P<0.001.


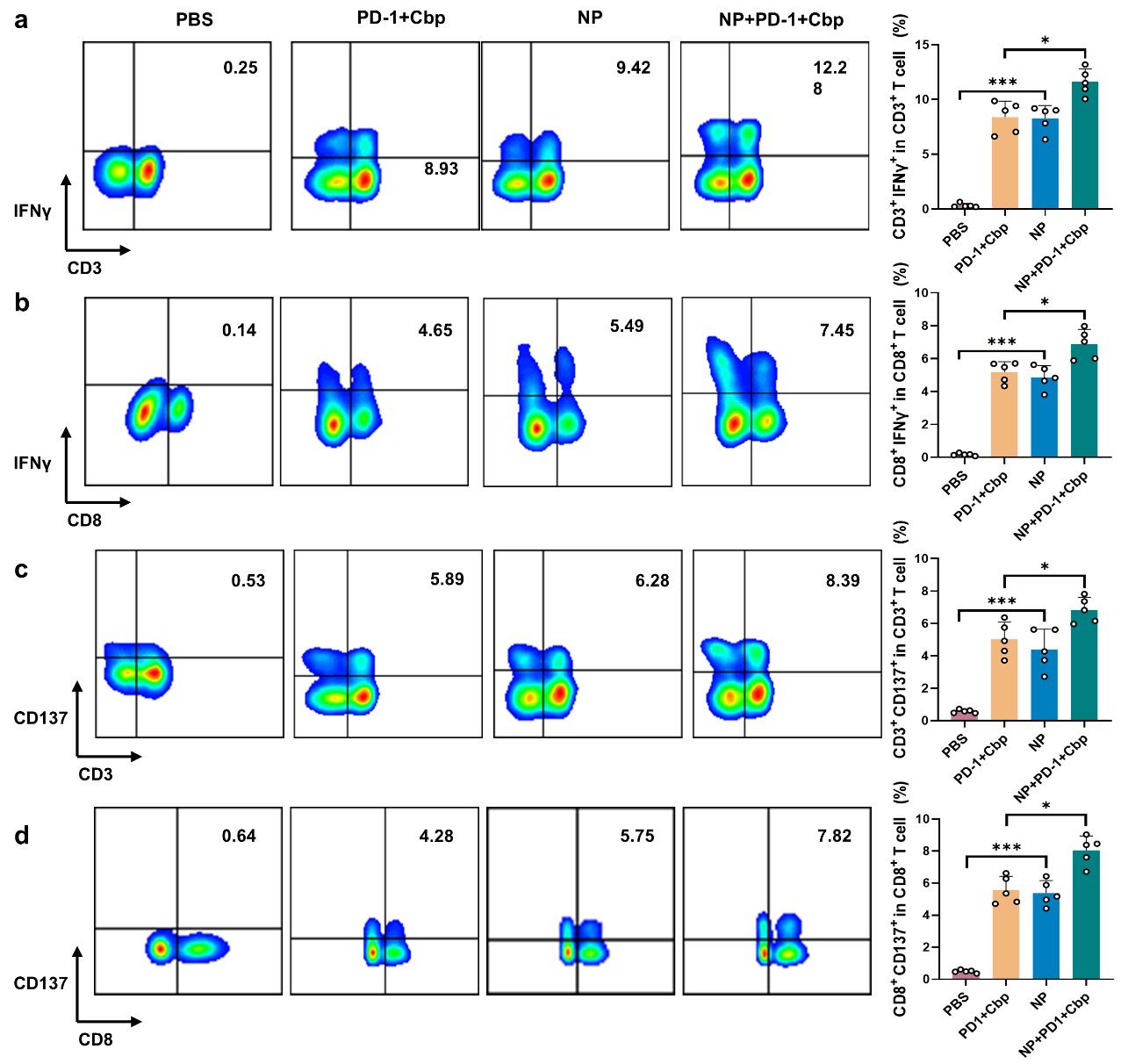


**Supplementary Fig. S6 Antigen NPs induced an increase of ETASTs in peripheral and the amount of ETASTs is positively correlated with treatment efficacy. a**, Representative flow cytometry results of IFN-γ^+^ T cells (ETASTs) in each group (left) and summary (right). **b**, Representative flow cytometry results of IFN-γ^+^ CD8^+^ T cells (ETASTs) in each group (left) and summary (right). **c**, Representative flow cytometry results of CD 137^+^T cells (ETASTs) in each group (left panel) and summary (right). **d**, Representative flow cytometry results of CD8^+^CD137^+^ T cells (ETASTs) in each group (left) and summary (right). Data are presented as Mean ± SEM. P-values <0.05 were considered significant: ^*^P<0.05, ^**^P<0.01, ^***^P<0.001.


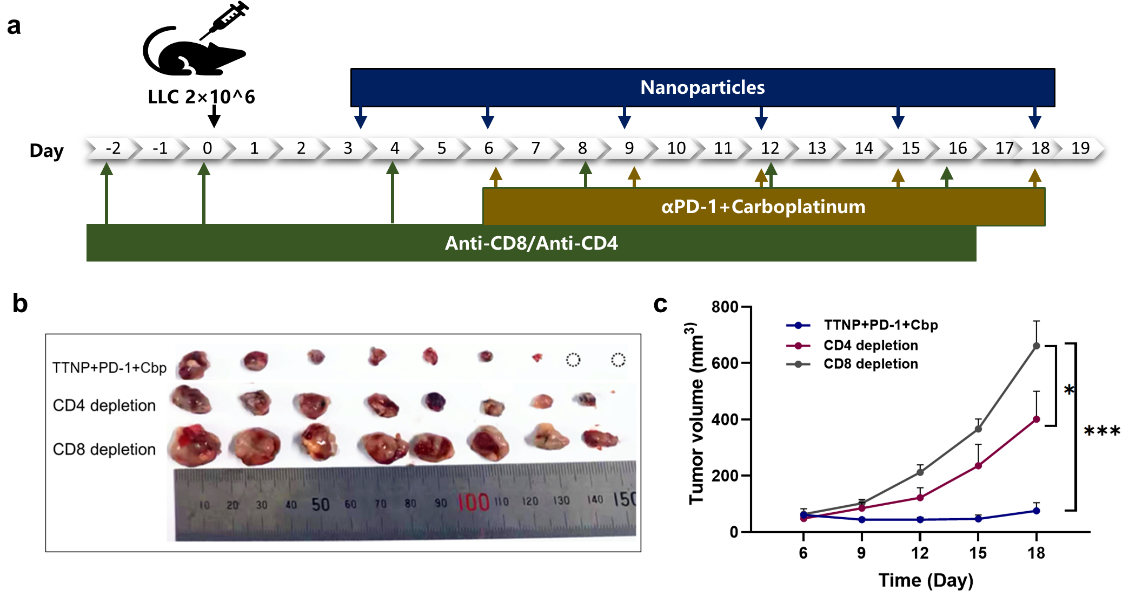


**Supplementary Fig.S7 Antigen NPs primarily activate CD8^+^ T cells to exert anti-tumor effects. a**, Tumor inoculation, depletion antibody and drug injection schedules in T-cell depletion experiments. **b**, Photographs of tumors in each group at the end of treatment. **c**, Tumor volume growth curves of mice in each group. Data are presented as Mean ± SEM. P-values <0.05 were considered significant: ^*^P<0.05, ^**^P<0.01, ^***^P<0.001.


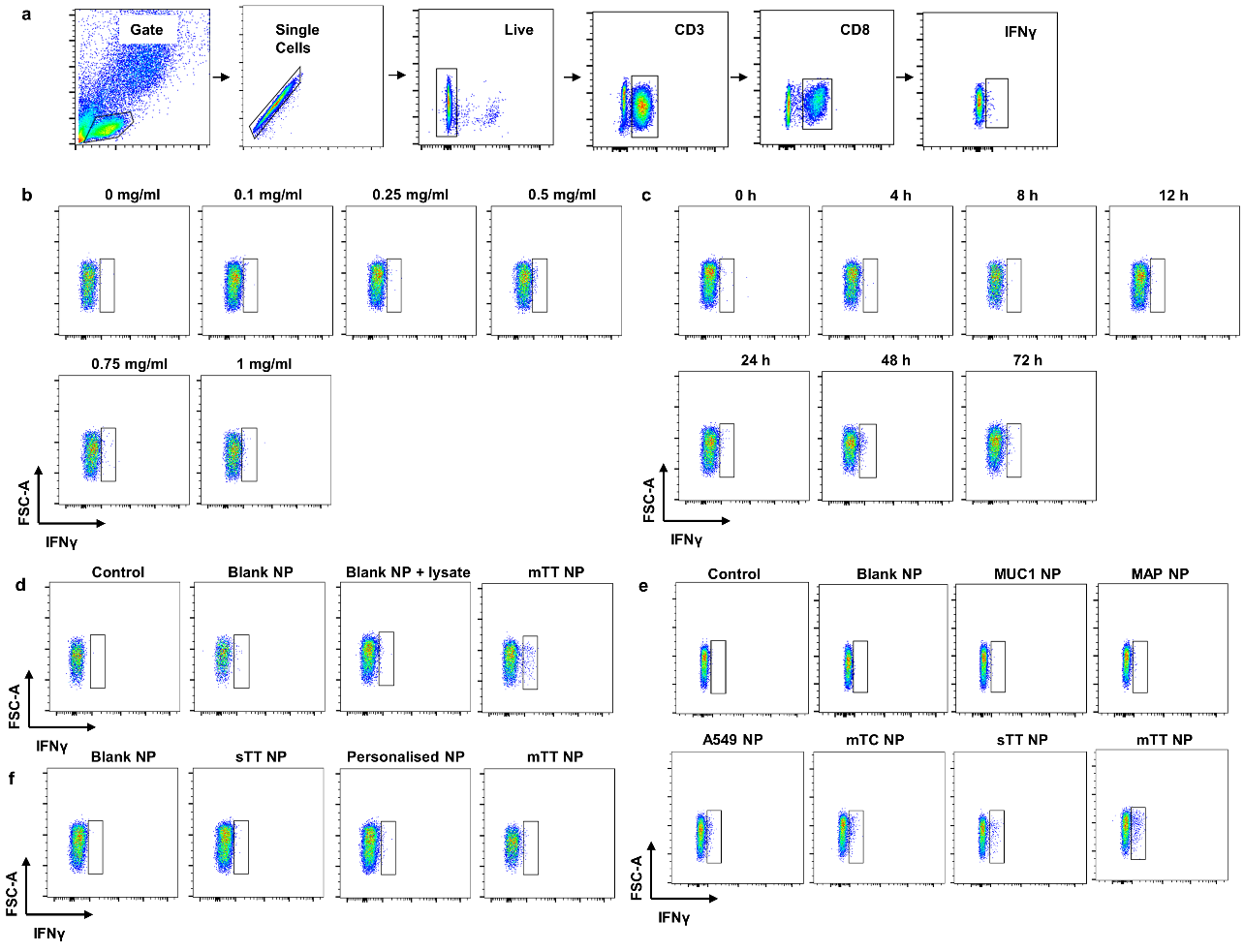


**Supplementary Fig. S8** **NPs loaded with whole cell antigens from different tumor tissues efficiently detect ETAST levels in the peripheral blood of NSCLC patients. a**, Flow cytometry plot showing the gating strategy of ETASTs activated by antigen nanoparticles in PBMC. **b**, Representative flow cytometry trsults of CD8^+^IFNγ^+^ T cells (ETASTs) activated by different nanoparticle concentrations. **c**, Representative flow cytometry results of CD8^+^IFNγ^+^ T cells (ETASTs) activated by different co-incubation times. **d**, Representative flow cytometry results of CD8^+^IFNγ^+^ T cells (ETASTs) activated by different antigen loading modalities. (**e**, **f**) Representative flow cytometry results of CD8^+^IFNγ^+^ T cells (ETASTs) activated by nanoparticles loaded with different kinds of antigens.


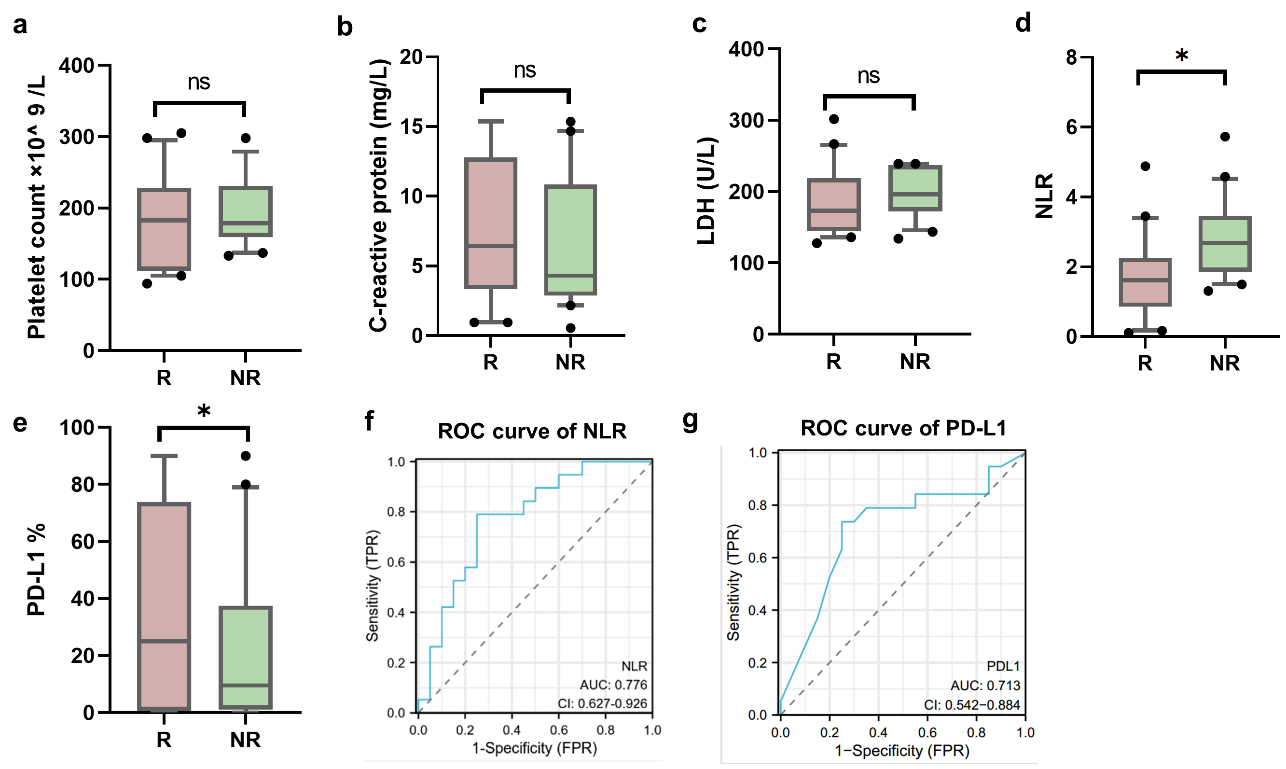


**Supplementary Fig. S9 Relationship between clinical indicators and the efficacy of chemoimmunotherapy combination. a**, Comparison of platelet counts in different response groups. **b**, Comparison of C-reactive protein levels in different response groups. **c,** Comparison of lactate dehydrogenase levels in different response groups. **d**, Comparison of NLR in different response groups. **e**, Comparison of PD-L1 expression in different response groups. **f**, ROC curve of NLR predicting the effect of chemoimmunotherapy in patients with NSCLC. **g**, ROC curve of PD-L1 expression predicting the effect of chemoimmunotherapy in patients with NSCLC. LDH, Lactate dehydrogenase; NLR, Neutrophil-lymphocyte ratio. n=40, data presented as Mean ± SEM, P-values <0.05 were considered significant: ^*^P<0.05. ns, not significant.


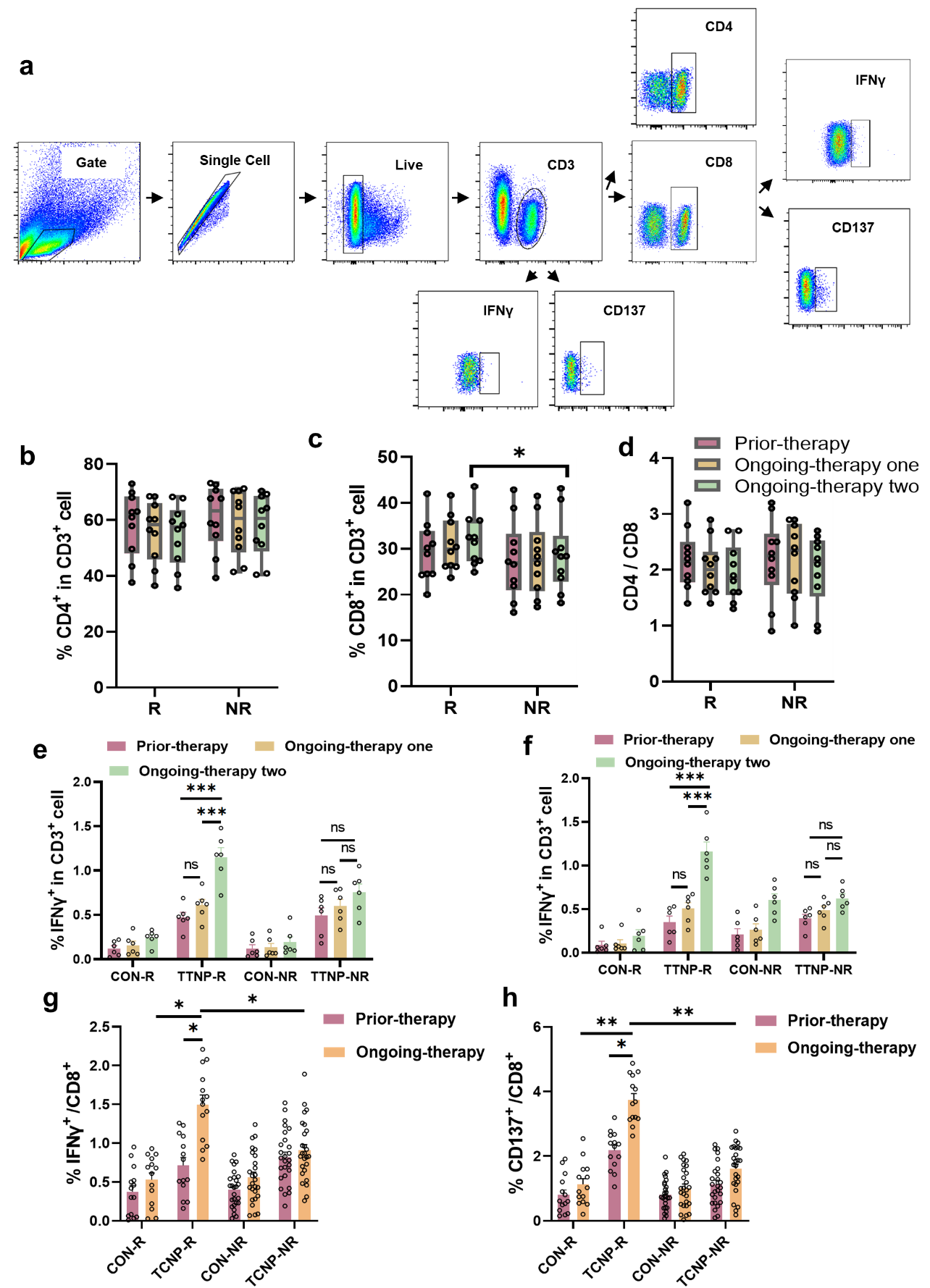


**Supplementary Fig. S10** **Changes in ETAST levels in peripheral blood of NSCLC patients receiving immunotherapy. a**, Flow cytometry results showing the gating strategy of ETASTs in PBMC. **b**, Changes in the levels of CD4^+^ T cells in blood across treatment cycles (n=20). **c**, Changes in the levels of CD8^+^ T cells in blood across treatment stages (n=20). **d**, CD4/CD8 in peripheral blood across treatment cycles (n=20). **e,** Changes in levels of CD3^+^IFN-γ^+^ T cells in peripheral blood after co-incubation with antigenic nanoparticles in NSCLC patients at various treatment cycles (n=12). **f**, Changes in levels of CD8^+^IFN-γ^+^ T cells in peripheral blood after co-incubation with antigenic nanoparticles in NSCLC patients at various treatment cycles (n=12). R, treatment responsive group; NR, treatment non-responsive group; CON, blank control group; TTNP, multi-tumor tissue antigen nanoparticles. Data are presented as Mean ± SEM. One date point represents one patient. P-values <0.05 were considered significant: ^*^P<0.05, ^**^P<0.01, ^***^P<0.001.


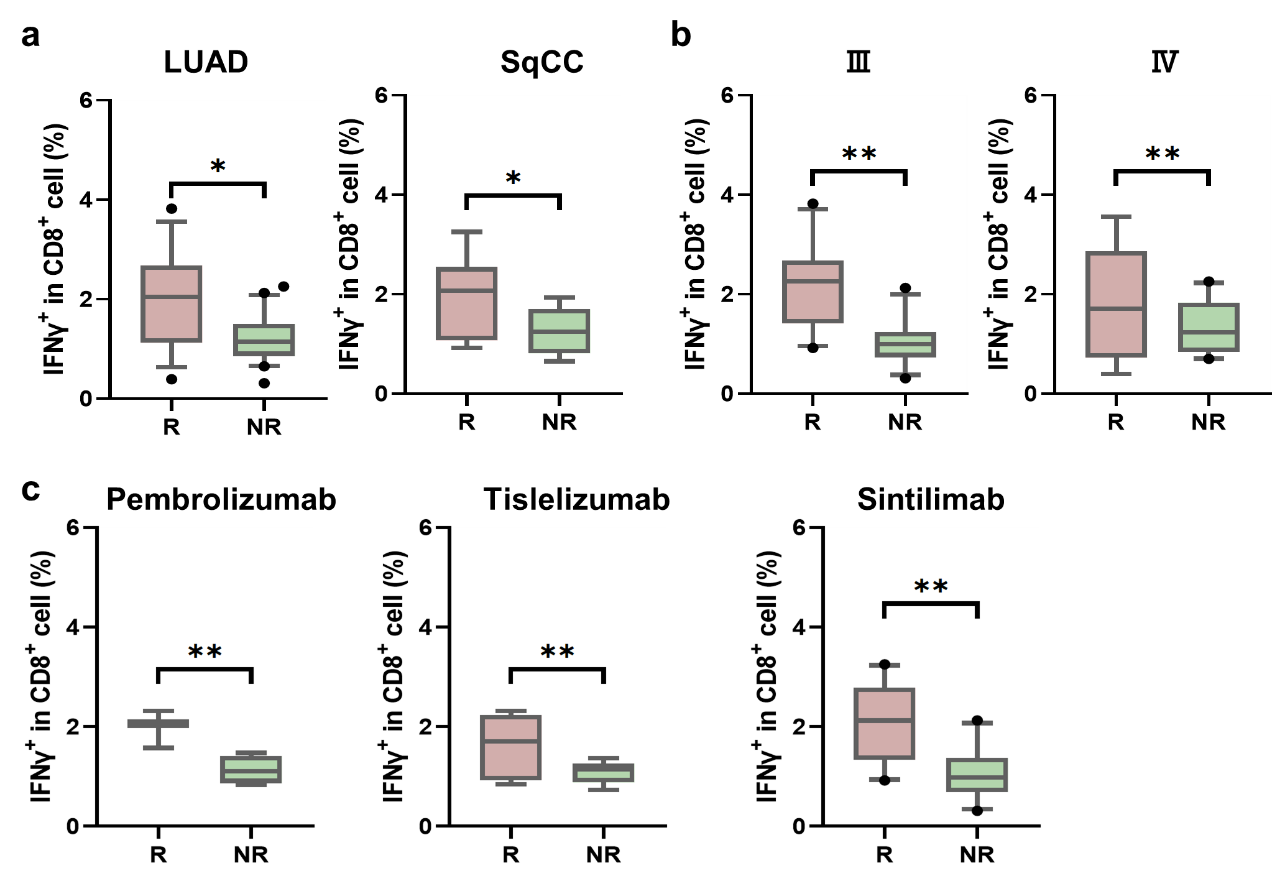


**Supplementary Fig. S11** **The relationship between the amount of ETASTs and pathological types, clinical staging, and the utilized immune checkpoint inhibitors. a**. The relationship between different pathological types (lung adenocarcinoma and lung squamous cell carcinoma) and the content of CD8^+^ IFN-γ^+^ T cells. **b**. The relationship between different clinical stages (stage III and stage IV) and the content of CD8^+^ IFN-γ^+^ T cells. **c**. The relationship between various immune checkpoint inhibitors (pembrolizumab, tislelizumab, sintilimab) and the content of CD8^+^ IFN-γ^+^ T cells.


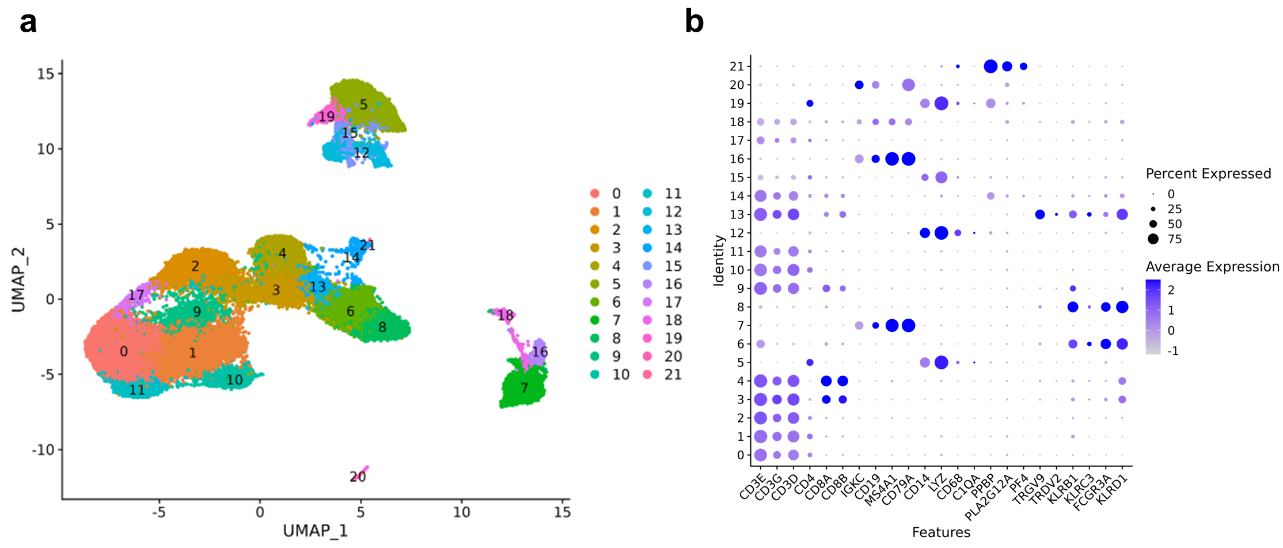


**Supplementary Fig. S12** **Single-cell sequencing characterization of PBMC from NSCLC patients**. **a**, 6 PBMC samples clustered to obtain 22 clusters of cells. **b**, Expression levels of marker genes in each cell cluster.
